# Psammaplysene D overcomes sorafenib resistance in liver cancer by targeting FGFR4/CYP26A1-retinoic acid axis to drive ferroptosis

**DOI:** 10.1186/s13046-025-03622-1

**Published:** 2026-01-06

**Authors:** Ting Yang, Yanlu Han, Yuting Wang, Ruyu Li, Xiaonan Zhang, Xinxin Zhang, Xiaohan Xu, Jing Xu, Xiaoyu Li, Chunhua Lin, Wen Wang, Jinbo Yang

**Affiliations:** 1 Key Laboratory of Marine Drugs, Ministry of Education of China, School of Medicine and Pharmacy, Ocean University of China, Qingdao, 266003 China; 2 Marine Biomedical Research Institute of Qingdao, Qingdao, 266071 China; 3 Department of Gastroenterology, The Affiliated Hospital of Qingdao University, Qingdao, China; 4 Department of Urology, the Affiliated Yantai Yuhuangding Hospital of Qingdao University, Yantai, Shandong 264000 China; 5 Qilu Hospital of Shandong University, Qingdao, Shandong 266000 China

**Keywords:** Liver cancer, Sorafenib resistance, Psammaplysene D, FGFR4, CYP26A1, Retinoic acid, Ferroptosis

## Abstract

**Background:**

Overcoming sorafenib resistance remains a major challenge in liver cancer treatment. This study evaluates the novel compound Psammaplysene D, alone or combined with sorafenib, against liver cancer, focusing on overcoming resistance.

**Methods:**

The efficacy of Psammaplysene D, alone or with sorafenib, was assessed using liver cancer cell lines and xenograft mouse models, including sorafenib-resistant variants. The direct binding interaction between Psammaplysene D and FGFR4 was confirmed through molecular docking and Cellular Thermal Shift Assay (CETSA). Transcriptomic profiling (RNA-seq) identified key differentially expressed genes. Ferroptosis induction was evaluated using key markers, and functional roles were validated using genetic and pharmacological approaches.

**Results:**

Psammaplysene D inhibited liver cancer growth in vitro and in vivo, alone or synergistically with sorafenib, and overcame sorafenib resistance in both models. Mechanistic investigations revealed that Psammaplysene D directly targets FGFR4, inducing ferroptosis. In sorafenib-resistant cells, Psammaplysene D downregulates CYP26A1 expression, elevating retinoic acid (RA) levels. FGFR4 inhibition triggered ferroptosis and reduced CYP26A1 expression, while accumulated RA drove ferroptosis in resistant cells.

**Conclusions:**

Overall, Psammaplysene D is a potent therapeutic agent for liver cancer, effective alone or combined with sorafenib, and overcomes resistance through direct targeting of FGFR4, initiating a cascade of CYP26A1 downregulation, RA accumulation, and ferroptosis induction-defining a novel FGFR4/CYP26A1/RA axis regulating ferroptosis in resistant liver cancer.

**Graphical Abstract:**

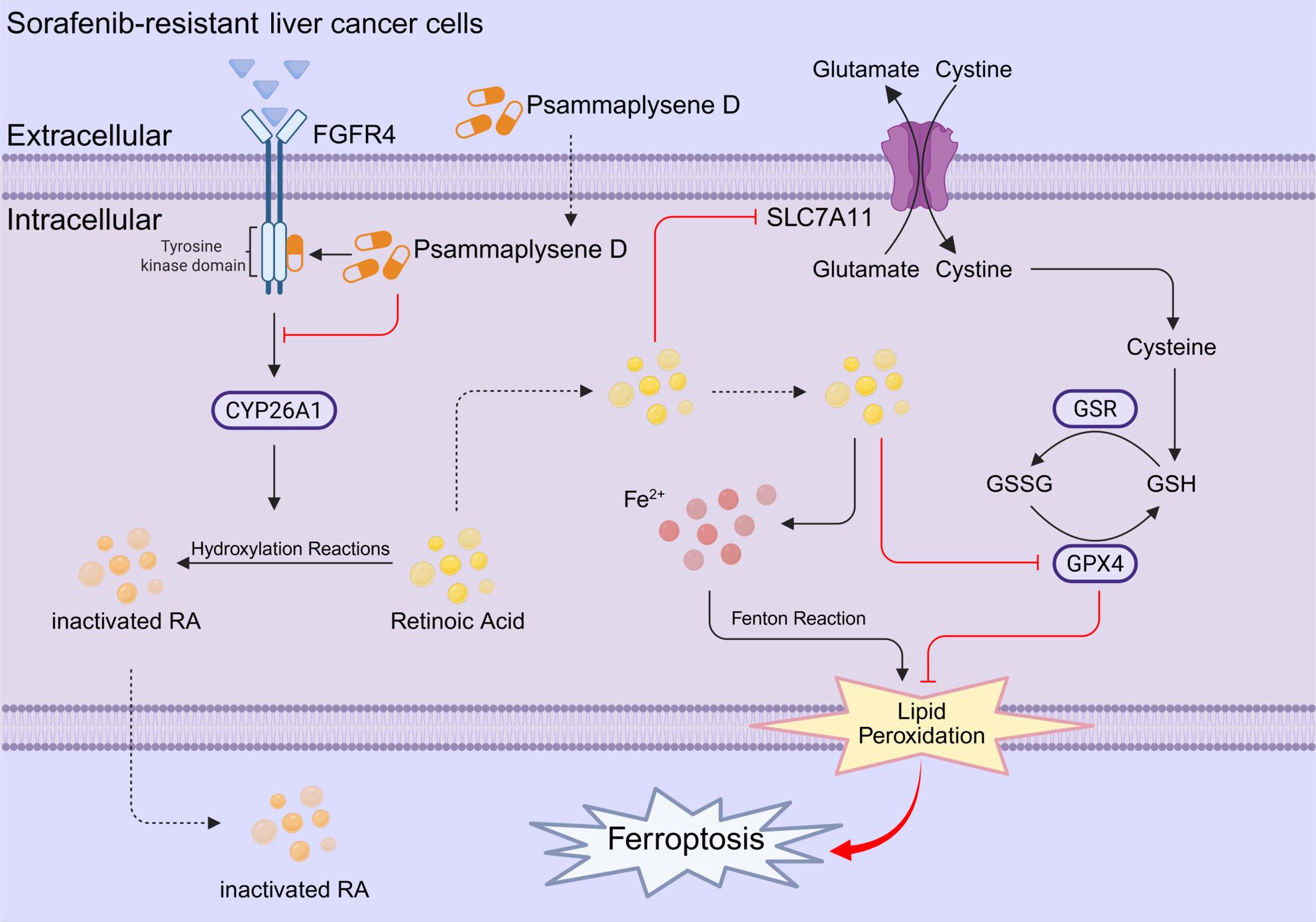

**Supplementary Information:**

The online version contains supplementary material available at 10.1186/s13046-025-03622-1.

## Highlights

Psammaplysene D overcomes sorafenib resistance in liver cancer by inducing ferroptosis through targeting FGFR4.

Psammaplysene D synergizes with sorafenib, offering a novel therapeutic strategy for liver cancer treatment.

The CYP26A1-retinoic acid axis serves as a driver of ferroptosis in drug-resistant cancers.

FGFR4 inhibition suppresses CYP26A1 transcription, elevating retinoic acid levels to trigger lethal lipid peroxidation.

**Supplementary Information:**

The online version contains supplementary material available at 10.1186/s13046-025-03622-1.

## Introduction

Liver cancer is the third leading cause of cancer-related deaths worldwide, claiming approximately 830,000 lives annually [1]. The rising incidence of liver cancer is primarily driven by hepatitis B infection and metabolic diseases [2]. Over 60% of patients are diagnosed at an advanced stage of the disease that is inoperable, resulting in a five-year survival rate of less than 10% for advanced liver cancer [3, 4]. Sorafenib remains a foundational systemic therapy; however, its efficacy is limited by a low response rate [5, 6]. This significant treatment gap underscores the urgent need to develop innovative molecularly targeted drugs and optimize combination therapy strategies to improve survival outcomes.

Since 2007, sorafenib has served as the first-line systemic therapy for advanced liver cancer. As a multi-kinase inhibitor targeting the VEGFR, PDGFR, and RAF pathways, it extends median overall survival by 2–3 months through dual inhibition of tumor angiogenesis and proliferation [7]. However, a portion of patients still develop acquired resistance within a few months, primarily driven by intrinsic adaptive changes in the tumor [8]. Sorafenib resistance has become an obstacle to prolonging overall survival in liver cancer patients. Therefore, overcoming this resistance remains an urgent need to lengthen the survival of patients with advanced liver cancer.

Fibroblast growth factor receptor 4 (FGFR4) belongs to the FGF receptor family and is predominantly expressed in the liver [9]. FGFR4 plays a broad role in metabolism and energy homeostasis [10], such as regulating bile acid synthesis [11], gluconeogenesis [12], and glycogen synthesis [13]. In liver cancer, high expression of FGFR4 is closely associated with liver cancer progression [9, 14]. It has been reported that abnormal activation of FGFR4 can enhance the metastatic capacity of liver cancer through the GSK3β-β-catenin signaling pathway [15]. This suggests that FGFR4 is a promising therapeutic target for liver cancer, and the development of small-molecule inhibitors targeting FGFR4 is a hot research direction [16]. Recently, two FGFR4-specific inhibitors, BLU9931 and FGF401, have been undergoing preclinical or clinical studies for liver cancer [9]. Furthermore, previous studies have reported that FGFR4 expression is increased in sorafenib-resistant liver cancer [17]. Using BLU9931 to inhibit FGFR4 activity can enhance the sensitivity of resistant tumors to sorafenib [18]. However, there are few reports on how the inhibition of FGFR4 affects sorafenib-resistant tumors.

**Fig. 1:**
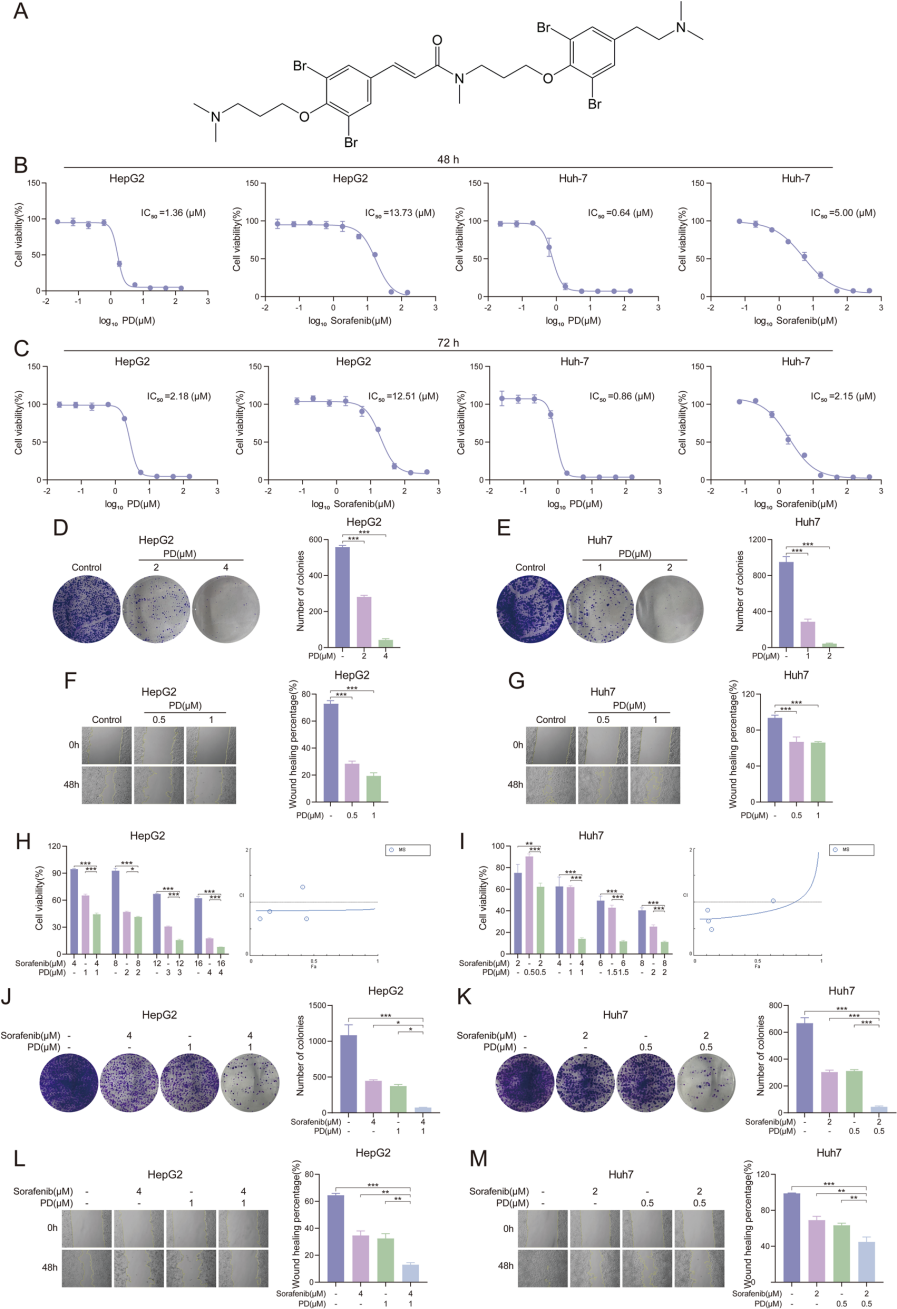
PD inhibits the growth of liver cancer cells alone or synergistically with sorafenib in vitro. **A** Chemical structure of PD. **B**-**G** HepG2 and Huh7 cells were treated with various concentrations of PD and sorafenib for the indicated time, and cell proliferation and migration were evaluated by **B**, **C**) MTT assay (*n* = 3), **D**, **E** colony formation assay (*n* = 3) and **F**, **G**) Wound Healing Assay (*n* = 3). **H**, **I** HepG2 and Huh7 cells were treated with various concentrations of PD and sorafenib separately or in combination for 48 h, and the combination index was calculated using Compusy software. **J**, **K** HepG2 and Huh7 cells were treated with various concentrations of PD and sorafenib separately or in combination for 48 h, followed by replacement with fresh medium and continued culture for 10 days, and cell proliferation was evaluated by colony formation assay (*n* = 3). **L**, **M**) HepG2 and Huh7 cells were treated with various concentrations of PD and sorafenib separately or in combination for 48 h, and migration was evaluated by Wound Healing Assay (*n* = 3). Data are presented as mean ± SEM. The *p*-values were calculated by one-way ANOVAs. *P < 0.05, **P < 0.01, and ***P < 0.001

Psammaplysene D (PD) (Fig. 1A) was first isolated from the marine sponge *Psammoclemma sp.* in 2007 and belongs to the class of bromotyrosine alkaloids [19]. Bromotyrosine alkaloids have been reported to exhibit a wide range of biological activities, including DNA demethylation [20], anti-proliferative [21], and antibacterial activities [22]. It has been reported that PD exhibits anti-proliferative and acetylcholinesterase inhibitory activities. PD exhibits an IC₅₀ of 1.3 µM for acetylcholinesterase inhibition. It also shows cytotoxicity against the human epidermoid carcinoma (KB) cell lines with an IC₅₀ of 0.7 µM [23]. This suggests that PD may be potentially effective in inhibiting the growth of other tumors, such as liver cancer.

Currently, combined therapy has been proven effective in the treatment of treatment-resistant liver cancer. As the combination of atezolizumab and bevacizumab can inhibit tumor growth and prolong survival, its efficacy is superior to that of either drug used alone [6]. Therefore, we sought to determine the therapeutic potential of PD in liver cancer and sorafenib-resistant liver cancer. A key focus was to address whether PD's activity is associated with FGFR4 and how it influences sorafenib resistance.

## Materials and methods

### Reagents

Anti-β-Actin (4967) was purchased from Cell Signaling Technology (Danvers, MA, USA). Anti-CYP26A1 (A5982), anti-GPX4 (A1933), and anti-SLC7A11 (A2413) were purchased from Abclonal (Wuhan, China). Lipid Peroxide (LPO) Content Assay Kit (BC5245), Crystal Violet Ammonium Oxalate Solution (G1062), and Thiazolyl blue tetrazolium bromide (MTT) (M8180) were purchased from Solarbio (Beijing, China). Alanine aminotransferase (ALT) Assay Kit (C009-2–1), Aspartate aminotransferase (AST) Assay Kit (C010-2–1), Cell Malondialdehyde (MDA) assay kit (A003-4–1), and Reduced glutathione (GSH) assay kit (A006-2–1) were purchased from Nanjing Jiancheng Bioengineering Institute (Nanjing, China). Cell Ferrous Iron Colorimetric Assay Kit (E-BC-K881-M) was purchased from Elabscience (Wuhan, China). Human Retinoic Acid ELISA Kit (CSB-E16712h) was purchased from CUSABIO (Wuhan, China). N-Nitrosodiethylamine (DEN) (E0136) and Sorafenib(S7397) were purchased from Selleck Chemicals (Houston, Texas, USA). BLU9931(T2347) was purchased from TargetMol (Boston, MA, USA). Retinoic Acid (HY-14649) was purchased from MCE (New Jersey, USA). PD was synthesized in the laboratory, and its structure was confirmed by NMR.

### Cell lines

Human liver cancer cell lines HepG2 and Huh7 were obtained from the American Type Culture Collection (ATCC). Cells were cultured in Dulbecco's Modified Eagle Medium (DMEM) containing 10% fetal bovine serum (FBS) and 1% penicillin/streptomycin. Cells were cultured in a humidified incubator at 37 °C with 5% CO₂. HepG2-SR and Huh7-SR cell lines were established through approximately five months of sorafenib gradient exposure to induce drug resistance.

### Cell viability assay

HepG2/HepG2-SR (12,000 cells/well) or Huh7/Huh7-SR (5,000 cells/well) were seeded into 96-well plates and cultured for 12 h. Cells were treated with either solvent or compound. After 48 h, add 20 μL of MTT (5 mg/mL) to each well, and continue incubating the cells for 2–4 h. The medium was then removed, and the precipitate was dissolved in DMSO. Absorbance was measured at 490 nm using a SpectraMax® i3, and cell viability was calculated.

### Colony-formation assay

HepG2/HepG2-SR (3,000 cells/well) or Huh7/Huh7-SR (2,000 cells/well) were seeded into 6-well plates containing 2 mL of complete medium. After 12 h, the cells were treated with the compounds or DMSO for 48 h, followed by replacement with fresh medium and continued culture for 10 days. At the end of the culture period, cells were washed with PBS, fixed with methanol, and stained with 0.2% crystal violet. Images were captured and analyzed after staining.

### RA ELISA assay

The concentration of retinoic acid (RA) was measured according to the manufacturer's protocol for the RA ELISA kit. This kit has a defined measurement range from 6.25 pg/ml to 100 pg/ml and a sensitivity of 3.12 pg/ml. Each sample was analyzed in three or more independent biological replicates.

### Wound healing assay

HepG2 or Huh7 cells were seeded at a density of 2 × 10^4^ cells per well in Culture-Insert 2 Well (Cat. No. 80206, ibidi, Germany). After 12 h of culture, the cells were further cultured for 48 h using a serum-free medium containing the compounds. Photographs were taken, and data were recorded at 0 h and 48 h.

### Protein lysate preparation and western blotting

Cells were washed with cold PBS and lysed on ice for 30 min using a cell lysis buffer containing 1 × protease inhibitor and 1 × phosphatase inhibitor. The cell lysate was then centrifuged at 12,000 × rcf for 10 min at 4 °C. Protein concentration was determined using the BCA method. The protein lysate was separated by SDS-PAGE and transferred to a PVDF membrane. The membrane was then blocked with 5% non-fat milk in TBST and incubated overnight at 4 °C with diluted primary antibody. After washing off the unbound primary antibody, the membrane was incubated with horseradish peroxidase (HRP)-labeled secondary antibody at room temperature for 2 h. Finally, the target protein was detected using a chemiluminescent HRP substrate, and images were captured using the Tanon 5200 chemiluminescent imaging system.

### Cellular Thermal Shift Assay (CETSA)

HepG2-SR cells were cultured in 10 cm culture dishes and collected when confluence reached 90%. After collection, the cells were washed twice with PBS, then collected in 1 mL of PBS, and subjected to three freeze–thaw cycles in liquid nitrogen. The supernatant was divided into two groups: DMSO and PD (20 μM), and incubated at 37 °C for 2 h. After incubation, the solution was aliquoted into 0.2 mL PCR tubes. Each tube was heated at the specified temperature for 3 min and then cooled to room temperature. Centrifuge at 12,000 × g for 10 min at 4 °C, then mix with the loading buffer. Finally, the samples are analyzed by Western blot.

### Plasmids, siRNA, shRNA, and transfection

All transfection operations were performed at a specified siRNA (GenePharma) concentration of 150 nM using Lipofectamine™ 3000 transfection reagent (Invitrogen). Plasmids and shRNA were transfected using Lipofectamine™ 3000 transfection reagent. Transfected cells were used for detection after being cultured for at least 48 h. The sequences are detailed in Supplementary Table 1–3.

### Isolation of RNA and qRT-PCR

Total RNA was purified using the RNA Rapid Purification Kit (ES Science, Shanghai, RN001) according to the manufacturer's instructions. Subsequently, RNA was reverse transcribed into cDNA using the PrimeScript™ RT Kit containing gDNA Eraser. cDNA was amplified using SYBR Green on the StepOne Plus Real-Time PCR System (Applied Biosystems). The target gene was normalized using β-actin as the internal reference gene. mRNA levels were calculated by comparing Ct values. Primer pair information is detailed in Supplementary Table 4.

### Animals

Six-week-old male BALB/c-nu mice and two-week-old male C57BL/6 mice were purchased from Beijing Vital River Laboratory Animal Technology Co., Ltd. (Beijing, China). All mice were housed in a specific pathogen-free (SPF) animal experimentation center maintained at a constant temperature, humidity, and 12-h light–dark cycle. All animal experiments were approved by the Animal Experimentation Committee of the Medical College of Marine Biomedical Research Institute of Qingdao (E-MBPD-2020–1-1).

#### Den-CCl_4_-induced liver cancer mouse model

Two-week-old mice were first administered a single intraperitoneal injection of DEN (25 mg/kg), followed by repeated administration of CCl₄ (25%) twice weekly starting at six weeks of age, continuing until 18 weeks of age. The control group received concurrent injections of solvent. At 14 weeks of age, mice injected with CCl₄ were randomly divided into three groups: model group, sorafenib-10 mg/kg, and PD-10 mg/kg, administered via intraperitoneal injection once daily until 18 weeks of age [24, 25]. At the end of the experiment, mice were euthanized with carbon dioxide, and liver and serum samples were collected for subsequent analysis.

#### HepG2 Xenograft Tumor Model-PD treatment with sorafenib

Mice were subcutaneously injected with 5 × 10⁶ HepG2 cells suspended in PBS. When tumor volume reached 50–100 mm^3^, mice were randomly divided into five groups: control group (5% DMSO, 8% HS15, and 87% saline), PD-5 mg/kg, PD-10 mg/kg, sorafenib-10 mg/kg, and PD combined with sorafenib group (10 mg/kg). All mice received daily intraperitoneal injections for two weeks. Body weight and tumor volume were recorded every two days. Tumor volume was calculated using the formula: tumor volume = ½ length × width^2^ based on digital caliper measurements. At the end of the experiment, all mice were euthanized with carbon dioxide, and tumor tissues were harvested, weighed, photographed, and stored for subsequent experiments.

#### HepG2-SR Xenograft Tumor Model-PD treatment with sorafenib

Mice were subcutaneously injected with 5 × 10⁶ HepG2-SR cells suspended in PBS. The mice were randomly divided into four groups: control group, PD-5 mg/kg, PD-10 mg/kg, and sorafenib-10 mg/kg. All mice received intraperitoneal injections daily for two weeks.

#### HepG2-SR Xenograft Tumor Model-PD treatment with BLU9931

Mice were subcutaneously injected with 5 × 10⁶ HepG2-SR cells suspended in PBS. The mice were randomly divided into four groups: control group, BLU9931-10 mg/kg, PD-10 mg/kg, and PD combined with BLU9931-10 mg/kg. All mice received intraperitoneal injections daily for two weeks.

### RNA sequencing

Total RNA was extracted from cells using Trizol reagent (Takara). The RNA libraries were sequenced by OE Biotech, Inc., Shanghai, China. We are grateful to OE Biotech, Inc. (Shanghai, China) for assisting in sequencing and/or bioinformatics analysis. Bioinformatic analysis was performed using the OECloud tools at https://cloud.oebiotech.com/task/. The volcano map (or other graphics) was drawn based on the R (https://www.r-project.org/) on the OECloud platform (https://cloud.oebiotech.com/task/).

### Statistical analysis

Statistical significance analysis was performed using GraphPad Prism 8.0 software. Paired t-tests or one-way analysis of variance (ANOVA) were used to determine statistical significance. P values < 0.05 were considered statistically significant.

## Results

### PD inhibits the growth of liver cancer cells alone or synergistically with sorafenib in vitro

To investigate whether PD could inhibit the growth of liver cancer cells, we selected two liver cancer cell lines, HepG2 and Huh7. We also tested the effects of sorafenib on two types of liver cancer cells. MTT assays showed that both PD and sorafenib inhibited the viability of liver cancer cells at 48 h and 72 h (Fig. 1B and C). Clone formation assays were used to investigate the role of PD on the proliferative capacity of liver cancer cells. As shown in Fig. 1D and E, PD inhibited the proliferation of both liver cancer cells in a dose-dependent manner. Meanwhile, PD inhibited the migration of liver cancer cells at the doses of 0.5 μM and 1 μM (Fig. 1F and G). These results indicated that PD was able to inhibit the development of liver cancer in vitro. Further, we investigated whether the combination of PD and sorafenib would have an additive effect on liver cancer cells. HepG2 and Huh7 cell lines were treated with PD and sorafenib alone or in a combination of both for 48 h, and cell viability was measured by MTT assay. We compared the effect of the combination treatment with that of PD or sorafenib alone and calculated the combination index (CI) using Compusy software [26]. The results demonstrated that the combination treatment of both PD and sorafenib significantly reduced cell viability compared with either treatment alone (Fig. 1H and I). When the CI < 1, it indicated that the combination of PD and sorafenib had a synergistic effect in inhibiting proliferation (Fig. 1H and I). Clone formation assay (Fig. 1J and K) and scratch assay (Fig. 1L and M) were used again to verify the effect of the co-administration on the proliferation and migration of liver cancer cells. As shown, the combination of PD and sorafenib was able to more significantly inhibit the proliferative and migratory abilities of HepG2 and Huh7 cell lines. These results demonstrate that PD has potential antitumor effects in vitro and can synergize with sorafenib to inhibit liver cancer.

### PD alone or in synergy with sorafenib inhibits liver cancer cell growth in vivo

**Fig. 2:**
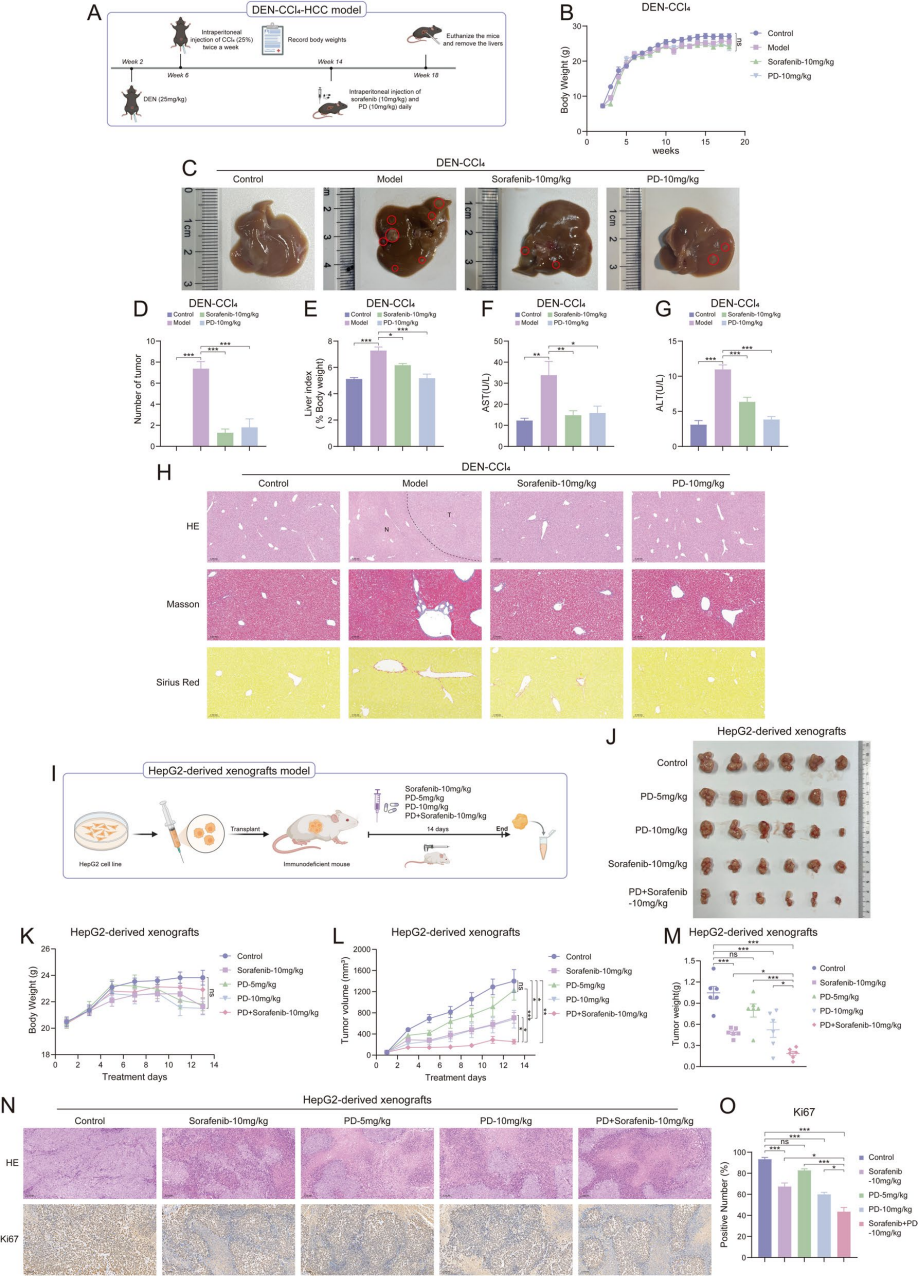
PD alone or in synergy with sorafenib inhibits liver cancer cell growth in vivo. **A** Schematic diagram of the DEN-CCl_4_-liver cancer model. **B**-**H** Mice were intraperitoneally administered PD (10 mg/kg) and sorafenib (10 mg/kg) every day, *n* = 5. B) body weight, **C** representative images of the livers, **D** the number of tumors, **E** liver index, **F** AST, **G** ALT, **H** representative images of hematoxylin and eosin (HE) (Scale bar, 200 μm), Masson (Scale bar, 100 μm) and Sirius Red (Scale bar, 100 μm) staining in liver tissues are shown (*n* = 5). **I** Schematic diagram of the HepG2-derived xenografts model. **J**-**O** Nude mice bearing HepG2-derived xenografts were intraperitoneally administered PD (5 mg/kg or 10 mg/kg) and sorafenib (10 mg/kg) every day, *n* = 6. J) representative images of the tumors, **K** body weight, **L** tumor growth curves, **M** tumor weight, **N** representative images of HE (Scale bar, 100 μm) and Ki67 (Scale bar, 100 μm) staining in tumor tissues, **O** positive number of Ki67 are shown (*n* = 6). Data are presented as mean ± SEM. The *p*-values were calculated by one-way ANOVAs. *P < 0.05, **P < 0.01, and ***P < 0.001, and ns, not significant

To investigate the anti-liver cancer effect of PD in vivo, we established a DEN + CCl_4_ model (Fig. 2A), which closely resembles the pathophysiological process of human liver cancer [27, 28]. Mice were treated with DEN (25 mg/kg) at week 2 and received intraperitoneal injections of 25% CCl_4_ as a tumor promoter at week 6, which lasted until week 18, thus establishing the liver cancer model. Starting at week 14, mice were administered PD (10 mg/kg) or sorafenib (10 mg/kg) daily until week 18. During the process of the experiment, body weights were recorded (Fig. 2B). The experimental results showed that sorafenib and PD inhibited tumor formation (Fig. 2C). The number of tumors in the liver (Fig. 2D) and the percentage of liver weight (Fig. 2E) were also significantly reduced after being treated with PD and sorafenib. Similarly, mice in the drug-treated group had significantly lower levels of aspartate aminotransferase (AST) (Fig. 2F) and alanine aminotransferase (ALT) (Fig. 2G) than mice in the model group. In addition, tumor formation was observed in the model group by H&E staining and was less in the drug-treated mice than in the model group (Fig. 2H upper). Masson staining (Fig. 2H middle) and Sirius Red staining (Fig. 2H lower) showed that PD and sorafenib reduced collagen deposition in the livers of mice. Given that PD exhibited anti-tumor ability in the DEN-CCl_4_ model, we further constructed a HepG2 cell-derived xenograft (CDX) model (Fig. 2I) to explore the in vivo anti-tumor ability of PD and whether it has a synergistic effect with sorafenib in vivo. After the xenograft tumor model of HepG2 cells was established, the tumor-bearing mice were subjected to different treatments, and body weights were recorded during the experiment (Fig. 2K). The results, as shown in Fig. 2J, showed that PD (5 mg/kg and 10 mg/kg) was able to inhibit tumor growth, where the effect of 10 mg/kg of PD was comparable to that of sorafenib (10 mg/kg). The combination of PD and sorafenib had a more significant inhibitory effect on tumor growth than PD and sorafenib alone (Fig. 2J). The same results were able to be observed in tumor volume and tumor weight (Fig. 2L and M). H&E staining showed that PD (5 mg/kg and 10 mg/kg) and sorafenib-treated tumors displayed extensive cell death (Fig. 2N upper). Furthermore, IHC staining showed that Ki67 expression was significantly reduced in PD (5 mg/kg and 10 mg/kg) and sorafenib-treated tumors, indicating that liver cancer cell proliferation was inhibited (Fig. 2N lower and O). These results demonstrate that PD exhibits anti-tumor ability in vivo and produces synergistic anti-tumor effects with sorafenib.

### PD inhibits the proliferation of sorafenib-resistant liver cancer cells in vitro and in vivo

**Fig. 3:**
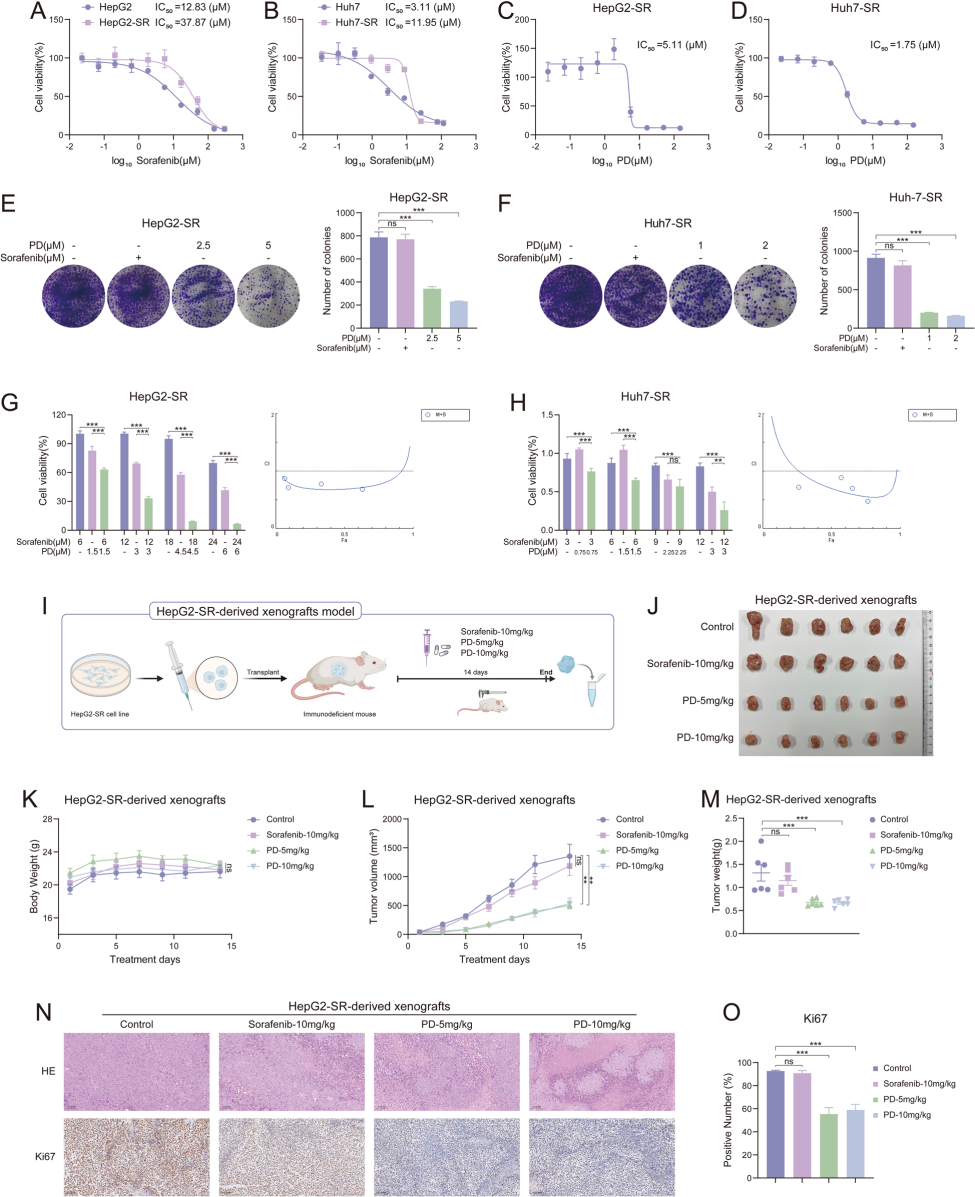
PD inhibits the proliferation of sorafenib-resistant liver cancer cells in vitro and in vivo. **A** HepG2 and HepG2-SR cells were treated with various concentrations of sorafenib for 48 h, and cell viability was evaluated by MTT assay (*n* = 3). **B** Huh7 and Huh7-SR cells were treated with various concentrations of sorafenib for 48 h, and cell viability was evaluated by MTT assay (*n* = 3). **C**, **D** HepG2-SR and Huh7-SR cells were treated with various concentrations of PD for 48 h, and cell viability was evaluated by MTT assay (*n* = 3). **E**, **F**) HepG2-SR and Huh7-SR cells were treated with various concentrations of PD and sorafenib for 48 h, followed by replacement with fresh medium and continued culture for 10 days, and cell proliferation was evaluated by colony formation assay (*n* = 3). **G**, **H** HepG2-SR and Huh7-SR cells were treated with various concentrations of PD and sorafenib separately or in combination for 48 h, and the combination index was calculated using Compusy software. **I** Schematic diagram of the HepG2-SR-derived xenografts model. **J**-**O** Nude mice bearing HepG2-SR-derived xenografts were intraperitoneally administered PD (5 mg/kg or 10 mg/kg) and sorafenib (10 mg/kg) every day, *n* = 6. **J** representative images of the tumors, **K** body weight, **L** tumor growth curves, **M** tumor weight, **N** representative images of HE (Scale bar, 100 μm) and Ki67 (Scale bar, 100 μm) staining in tumor tissues, **O** positive number of Ki67 are shown (*n* = 6). Data are presented as mean ± SEM. The *p*-values were calculated by one-way ANOVAs. **P < 0.01, and ***P < 0.001, and ns, not significant

To investigate the effect of PD on sorafenib-resistant liver cancer cells, we constructed sorafenib-resistant liver cancer cell lines (HepG2-SR and Huh7-SR) and measured the drug-resistant status of the cells. The IC_50_ curves showed that the HepG2-SR and Huh7-SR cells were more resistant to sorafenib than their parental cells (Fig. 3A and B). The MTT assays showed that PD was able to significantly inhibit the proliferation of sorafenib-resistant cells in a dose-dependent manner (Fig. 3C and D). In clone formation assays, we obtained consistent results that PD-treated cells had lower clone formation ability than the untreated and sorafenib-treated groups (Fig. 3E and F). In Fig. 1, we observed that PD exhibits synergistic effects with sorafenib in normal liver cancer cell lines. Therefore, we hypothesize that they may also produce synergistic effects in drug-resistant cell lines. HepG2-SR and Huh7-SR cell lines were treated with PD and sorafenib monotherapy or combination therapy for 48 h. Cell viability was assessed using the MTT assay, and the combination index (CI) was calculated using Compusy software. As shown in Fig. 3G and H, the combination treatment of both PD and sorafenib significantly reduced cell viability compared with either treatment alone. These results suggested that PD had an inhibitory effect on the growth of sorafenib-resistant tumors. To verify whether these in vitro experimental results could be confirmed in vivo, we injected HepG2-SR cells into immunodeficient mice to construct a xenograft tumor model (Fig. 3I). The mice were divided into four different treatment groups (control group, sorafenib-10 mg/kg, PD-5 mg/kg, and PD-10 mg/kg) and treated continuously for 14 days. Weight changes and tumor volume changes were monitored during the treatment period (Fig. 3K and L). There were no significant differences in body weight among the mice in the different treatment groups (Fig. 3K). The tumor volume in the sorafenib treatment group showed no significant difference compared to the control group, while PD effectively suppressed tumor growth (Fig. 3J and L). Interestingly, there was no significant difference in the therapeutic effects between the PD-5 mg/kg and PD-10 mg/kg dose groups, and no dose-dependent effect was observed (Fig. 3J and L). The results for tumor weight were consistent with the tumor volume data (Fig. 3M). To support these findings, tumor tissues were analyzed via hematoxylin and eosin (HE) staining and immunohistochemical staining, which revealed that PD induced tumor cell death and significantly reduced the expression of Ki67 (Fig. 3N and O). These results demonstrate that PD holds significant potential for treating sorafenib-resistant liver cancer.

### PD suppresses tumor growth by targeting FGFR4

**Fig. 4:**
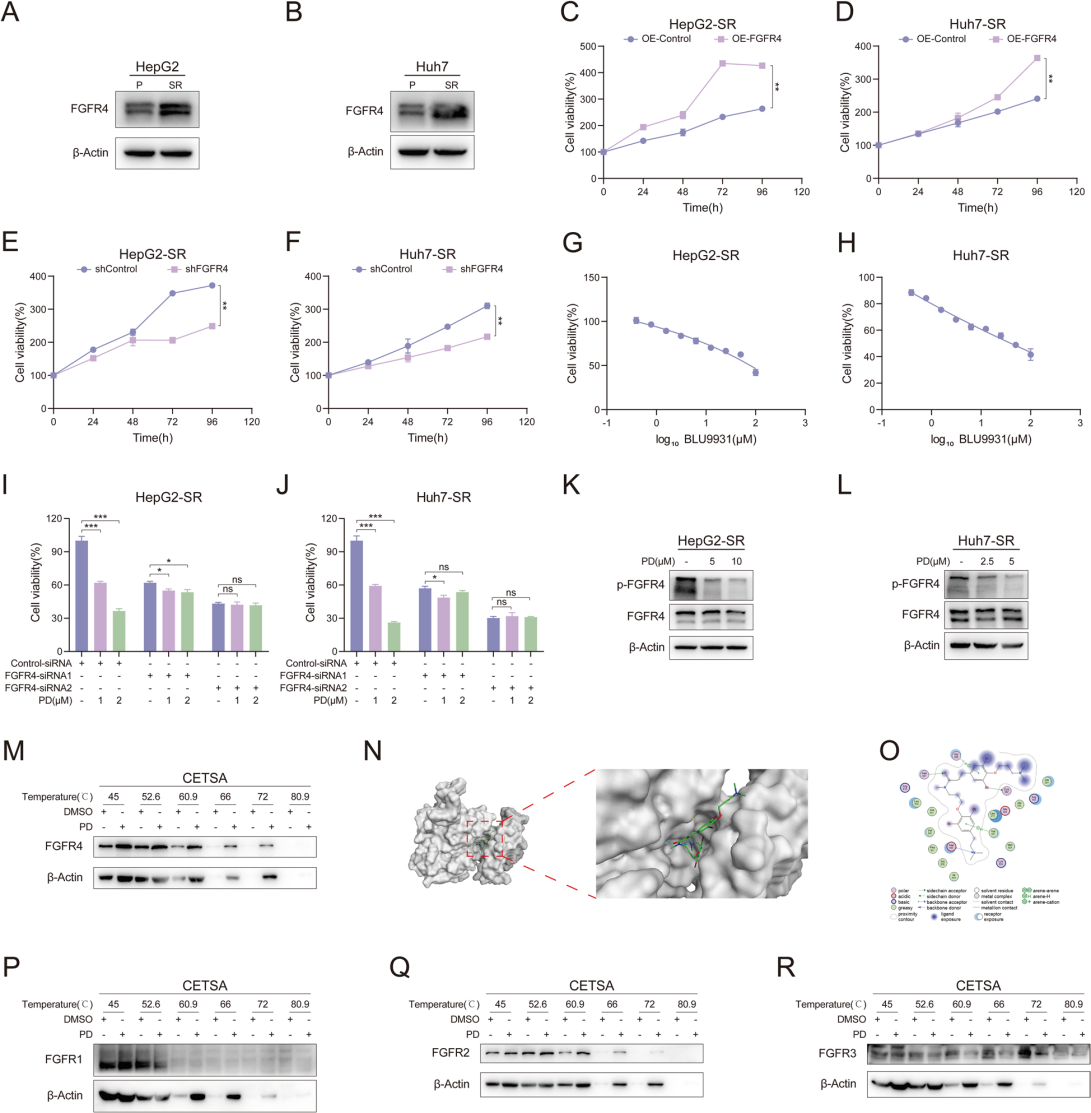
FGFR4 is the target of PD. **A**, **B** The expression levels of FGFR4 in drug-resistant cells and normal liver cancer cells. **C**, **D** MTT assay was carried out to examine the cell viability of **C**) HepG2-SR and **D**) Huh7-SR cells transfected with PLVX-puro-FGFR4 (*n* = 3). **E**, **F** Cell viability was examined in FGFR4-knockdown **E**) HepG2-SR and F) Huh7-SR cells by MTT assay (*n* = 3). **G**, **H** HepG2-SR and Huh7-SR cells were treated with various concentrations of BLU9931 for 48 h, and cell viability was evaluated by MTT assay (*n* = 3). **I**, **J** Cell viability was examined in FGFR4-knockdown **I**) HepG2-SR and **J**) Huh7-SR cells treated with PD by MTT assay (*n* = 3). **K**, **L** The expression levels of p-FGFR4 in drug-resistant cells after PD treatment. **M**) CETSA was carried out to evaluate the binding of PD with FGFR4. **N**, **O**) Molecular docking was performed to predict the binding region between PD and FGFR4, **N**) the docking configuration, and **O**) interaction forces are displayed. P-R) CETSA was carried out to evaluate the binding of PD with **P**) FGFR1, **Q**) FGFR2, and **R**) FGFR3. Data are presented as mean ± SEM. The p-values were calculated by one-way ANOVAs. *P < 0.05, **P < 0.01, and ***P < 0.001, and ns, not significant

**Fig. 5:**
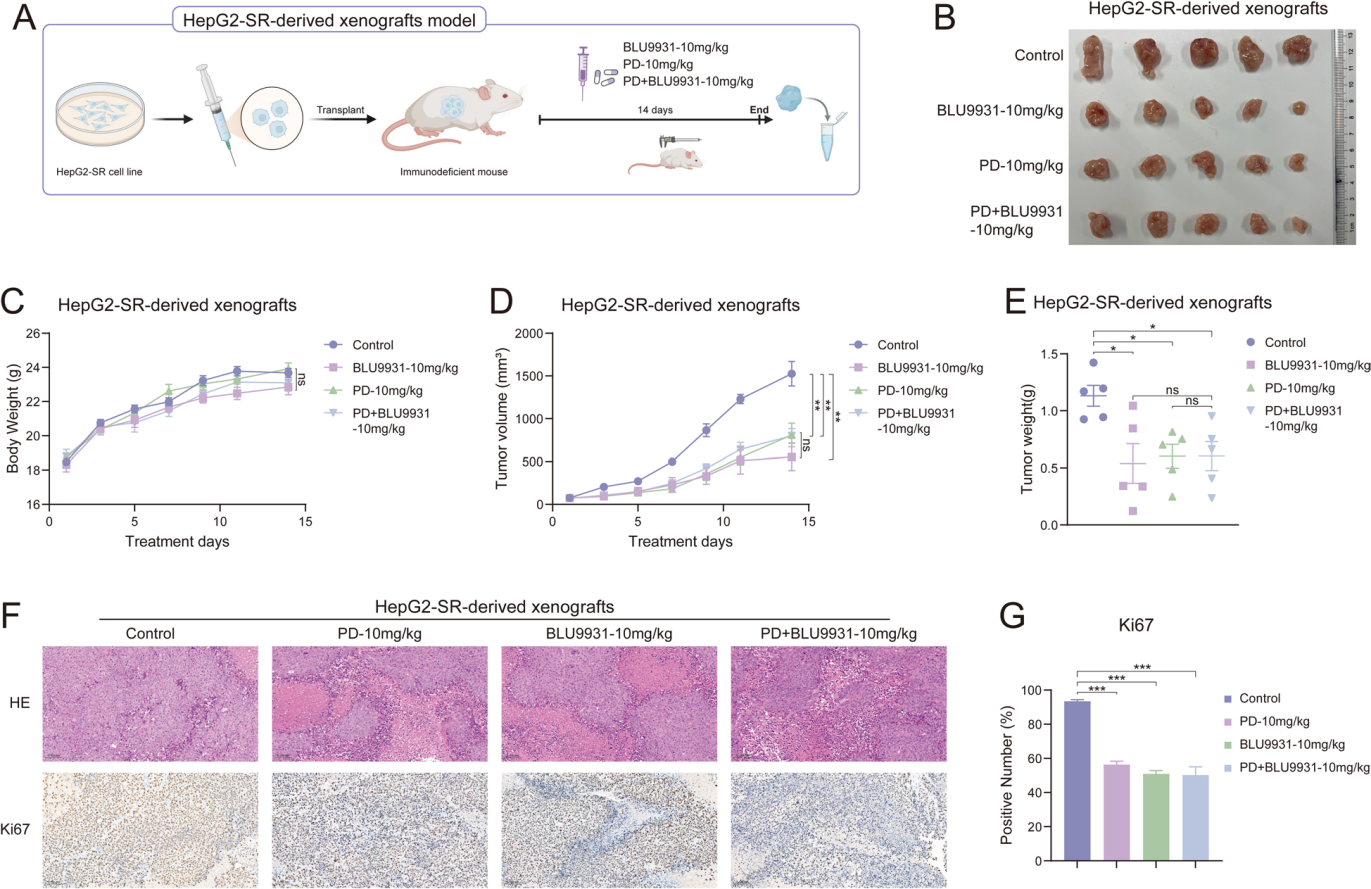
PD suppresses tumor growth by targeting FGFR4. **A** Schematic diagram of the HepG2-SR-derived xenografts model. **B**-**G** Nude mice bearing HepG2-SR-derived xenografts were intraperitoneally administered PD (10 mg/kg) and BLU9931 (10 mg/kg) every day, *n* = 5. **B** representative images of the tumors, **C** body weight, **D** tumor growth curves, **E** tumor weight, **F** representative images of HE (Scale bar, 100 μm) and Ki67 (Scale bar, 100 μm) staining in tumor tissues, **G** positive number of Ki67 (*n* = 5) are shown. Data are presented as mean ± SEM. The *p*-values were calculated by one-way ANOVAs. *P < 0.05, **P < 0.01, and ***P < 0.001, and ns, not significant

As a multi-targeted kinase inhibitor, sorafenib is a first-line treatment for advanced liver cancer (unresectable/metastatic), as well as for advanced renal cell carcinoma, radioactive iodine-refractory thyroid cancer, and acute myeloid leukemia with specific gene mutations (FLT3-ITD positive). Patients typically develop resistance after approximately one year, but in liver cancer patients, approximately 70% experience treatment failure or disease progression within six months of initial therapy (with significant individual variability, with some patients progressing as early as three to four months) [29, 30]. The resistance to sorafenib mainly stems from adaptive metabolic reprogramming and abnormal activation of signaling pathways in tumor cells, such as lipid metabolism disorders [31], continuous activation of survival pathways [32], and remodeling of the immune microenvironment [33]. At the same time, the strengthening of cellular defense mechanisms further exacerbates resistance, including improved antioxidant capacity [34] and epigenetic regulation [35]. Previous reports have indicated that FGFR4 expression is increased in sorafenib-resistant liver cancer cell lines [18]. Our experimental results also observed the same conclusion (Fig. 4A and B). Similarly, elevated FGFR4 expression also occurs in prolactinomas, gastric cancer, and cholangiocarcinoma [36–38]. Therefore, we first investigated whether regulating the expression of FGFR4 would affect the growth of sorafenib-resistant liver cancer cell lines. FGFR4 was overexpressed or knocked down in HepG2-SR and Huh7-SR cells, respectively, and cell viability was assessed by MTT assay. The efficiency of overexpression and knockdown were demonstrated in Supplementary Fig. 1. HepG2-SR and Huh7-SR cells grew more rapidly than the control group after FGFR4 overexpression (Fig. 4C and D). Meanwhile, the knockdown of FGFR4 inhibited the proliferation of these two drug-resistant cell lines (Fig. 4E and F). The pharmacological inhibition of FGFR4 is achievable through BLU9931, a specific inhibitor of FGFR4 [39, 40] (Fig. 4G and H). These results indicated that FGFR4 can promote the growth of sorafenib-resistant liver cancer cell lines, and FGFR4 may be a target for treating sorafenib-resistant liver cancer. Therefore, we examined the effect of PD on resistant cells when FGFR4 was knocked down. As expected, when FGFR4 was knocked down, the inhibitory effect of PD on the proliferation of HepG2-SR and Huh7-SR cells was partially offset (Fig. 4I and J). In the following experiments, we examined the effect of PD on the phosphorylation levels of FGFR4. As shown in Fig. 4K and L, PD significantly suppressed p-FGFR4 levels in both sorafenib-resistant cell lines. We consequently hypothesized that PD might bind to FGFR4. CETSA results confirmed that PD indeed interacted with FGFR4 (Fig. 4M). Furthermore, molecular docking simulations were conducted to model the conformation of PD binding to FGFR4 (PDB: 6jpe). As shown in Fig. 4N and O, PD can fit into the substrate-binding pocket of FGFR4 and interact with FGFR4 through hydrogen bonds formed with residues Valine 481 (Val481), Cysteine 552 (Cys552), Glycine 556 (Gly556), Asparagine 557 (Asn557), and Aspartate 630 (Asp630). Notably, Cys552 is a unique active pocket binding site specific of FGFR4 compared to other FGFR family members [41]. We also examined the binding capacity of PD with other members of the FGFR family (FGFR1, FGFR2, and FGFR3). The experimental results of CETSA are shown in Fig. 4P-R. PD has no significant effect on the thermal stability of FGFR1 (Fig. 4P) and FGFR3 (Fig. 4R). Although PD can enhance the thermal stability of FGFR2 (Fig. 4Q), the extent of enhancement was less pronounced than that observed for FGFR4. The molecular docking results (Supplementary Fig. 2) showed the binding regions of PD with FGFR1 (PDB: 5ew8), FGFR2 (PDB: 8e1x), and FGFR3 (PDB: 7dhl). The binding E-score between PD and FGFR4 was −8.47929573, slightly higher than the binding E-values with FGFR1 (E-score = −8.1158514), FGFR2 (E-score = −8.30552483), and FGFR3 (E-score = −8.39532185). These results suggested that PD inhibits sorafenib-resistant liver cancer through targeting FGFR4. This will be further confirmed by in vivo experiments. HepG2-SR cells were injected subcutaneously into nude mice to establish a xenograft tumor model (Fig. 5A). The mice were divided into four treatment groups: the control group, BLU9931-10 mg/kg, PD-10 mg/kg, and BLU9931 + PD-10 mg/kg. BLU9931 was used as a positive control. After 14 days of treatment, tumor tissues were collected and photographed (Fig. 5B). Tumor volumes in the BLU9931-10 mg/kg and PD-10 mg/kg treatment groups were significantly smaller than those in the control group, with no significant difference between the two groups (Fig. 5B and 5). Meanwhile, the tumor volumes in the group treated with the combination of BLU9931 and PD were slightly lower than those in the groups treated with either agent alone, but there were no statistically significant differences (Fig. 5B and D). There were also no significant differences in body weight among the mice in the various groups (Fig. 5C). The results for tumor weight also matched those for tumor volume (Fig. 5E). Hematoxylin and eosin (HE) staining (Fig. 5F upper) and immunohistochemical staining for Ki67 (Fig. 5F lower and G) further confirmed that PD and BLU9931 inhibited tumor growth, with results comparable to those of the group treated with the combination of BLU9931 and PD. These results indicate that when FGFR4 is pharmacologically inhibited, PD cannot exert its antitumor effects. In summary, the antitumor activity of PD was unchanged or partially offset when FGFR4 was inhibited pharmacologically or genetically, indicating that PD exerts its antitumor effect by targeting FGFR4 in sorafenib-resistant liver cancer.

### PD inhibits liver cancer progression by inducing ferroptosis

**Fig. 6:**
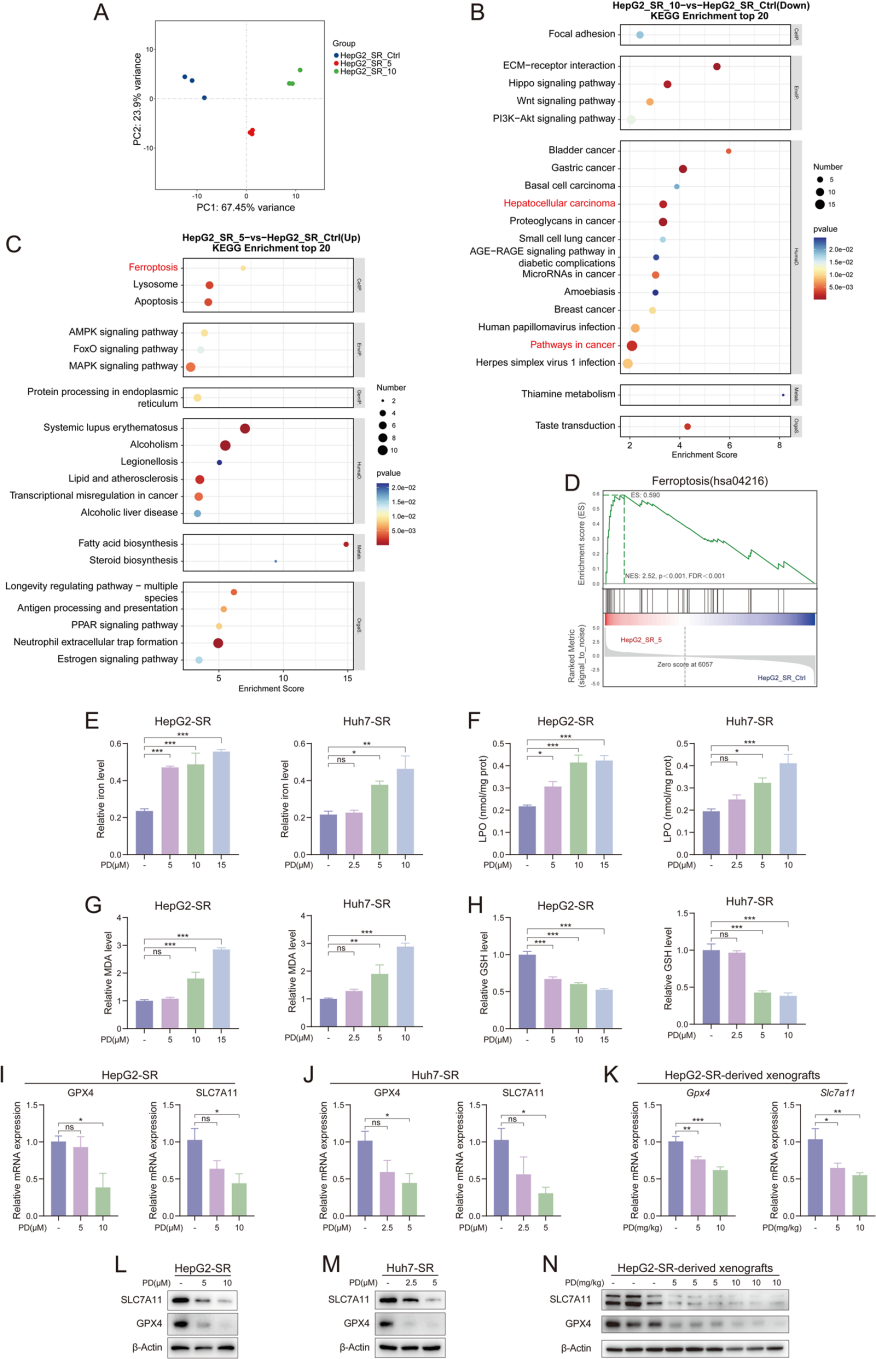
PD induces ferroptosis in sorafenib-resistant liver cancer cells. **A** PCA showing clustering of HepG2-SR-Ctrl (blue), HepG2-SR-5 (red), HepG2-SR-10 (green) along PC1 (67.45%)/PC2 (23.9%). **B** KEGG enrichment analysis of downregulated genes in HepG2-SR-10 compared with HepG2-SR-Ctrl from RNA-seq data. **C** KEGG enrichment analysis of upregulated genes in HepG2-SR-5 compared with HepG2-SR-Ctrl from RNA-seq data. **D** GSEA of the transcriptional profiles of HepG2-SR-5 and HepG2-SR- Ctrl with the genes related to ferroptosis. **E**-**J** HepG2-SR and Huh7-SR cells were treated with PD (2.5, 5, 10, and 15 μM) for 48 h. **E** iron level, **F** LPO level, **G** MDA level, **H** GSH, and **I**, **J**) relative mRNA levels of GPX4 and SLC7A11 were measured. **K** mRNA levels of Gpx2 and Slc7a11 in HepG2-SR-derived xenograft tissues (*n* = 6). **L**-**N** The protein levels of GPX4 and SLC7A11 in **L**) HepG2-SR, **M**) Huh7-SR cells, and **N**) HepG2-SR-derived xenograft tissues were measured by western blotting. Data are presented as mean ± SEM. The *p*-values were calculated by one-way ANOVAs. *P < 0.05, **P < 0.01, and ***P < 0.001, and ns, not significant

To elucidate the molecular mechanisms and signaling pathways by which PD inhibits liver cancer progression, we performed RNA sequencing on HepG2-SR cells treated with PD at 5 μM and 10 μM, as well as the control group, to detect changes in gene expression profiles and signaling pathways. Principal component analysis revealed significant differences in gene expression profiles among the control group, PD 5 μM-treated, and PD 10 μM-treated HepG2-SR cells (Fig. 6A). Kyoto Encyclopedia of Genes and Genomes (KEGG) analysis of these differentially expressed genes showed enrichment of pathways associated with liver cancer development and ferroptosis (Fig. 6B and C). Alterations in the ferroptosis process were also validated via gene set enrichment analysis (GSEA) (Fig. 6D). Ferroptosis is an iron-dependent form of cell death caused by excessive accumulation of lipid peroxides within cells. This process is catalyzed by iron ions and accompanied by the collapse of the cellular antioxidant system (such as glutathione peroxidase). Ferroptosis differs significantly from other forms of cell death, such as apoptosis and necrosis, in terms of morphology and mechanism, and is associated with various pathophysiological processes, including tumor suppression [42]. To confirm whether PD induces ferroptosis in sorafenib-resistant liver cancer cell lines, we measured changes in intracellular ferroptosis-related markers following PD treatment. The results showed that PD increased the levels of divalent iron ions (Fe^2+^) in HepG2-SR and Huh7-SR cells (Fig. 6E). Free iron ions can mediate lipid peroxidation, which is a key process in ferroptosis [43]. As shown in Fig. 6, after PD treatment, the levels of lipid peroxidation (LPO) and malondialdehyde (MDA) significantly increased in HepG2-SR and Huh7-SR cells (Fig. 6F and G), while the level of glutathione (GSH) showed a significant decrease (Fig. 6H). Furthermore, we examined changes in glutathione peroxidase 4 (GPX4) and solute carrier family 7a member 11 (SLC7A11), which are key genes associated with ferroptosis [43]. At the mRNA level, PD markedly downregulated the expression of GPX4 and SLC7A11 in HepG2-SR and Huh7-SR cells (Fig. 6I and J). The results were also observed through western blot analysis (Fig. 6L and M). Meanwhile, we also detected the expressions of GPX4 and SLC7A11 in HepG2-SR xenograft tumor tissues (Fig. 6K and N), which were consistent with those in vitro.

**Fig. 7:**
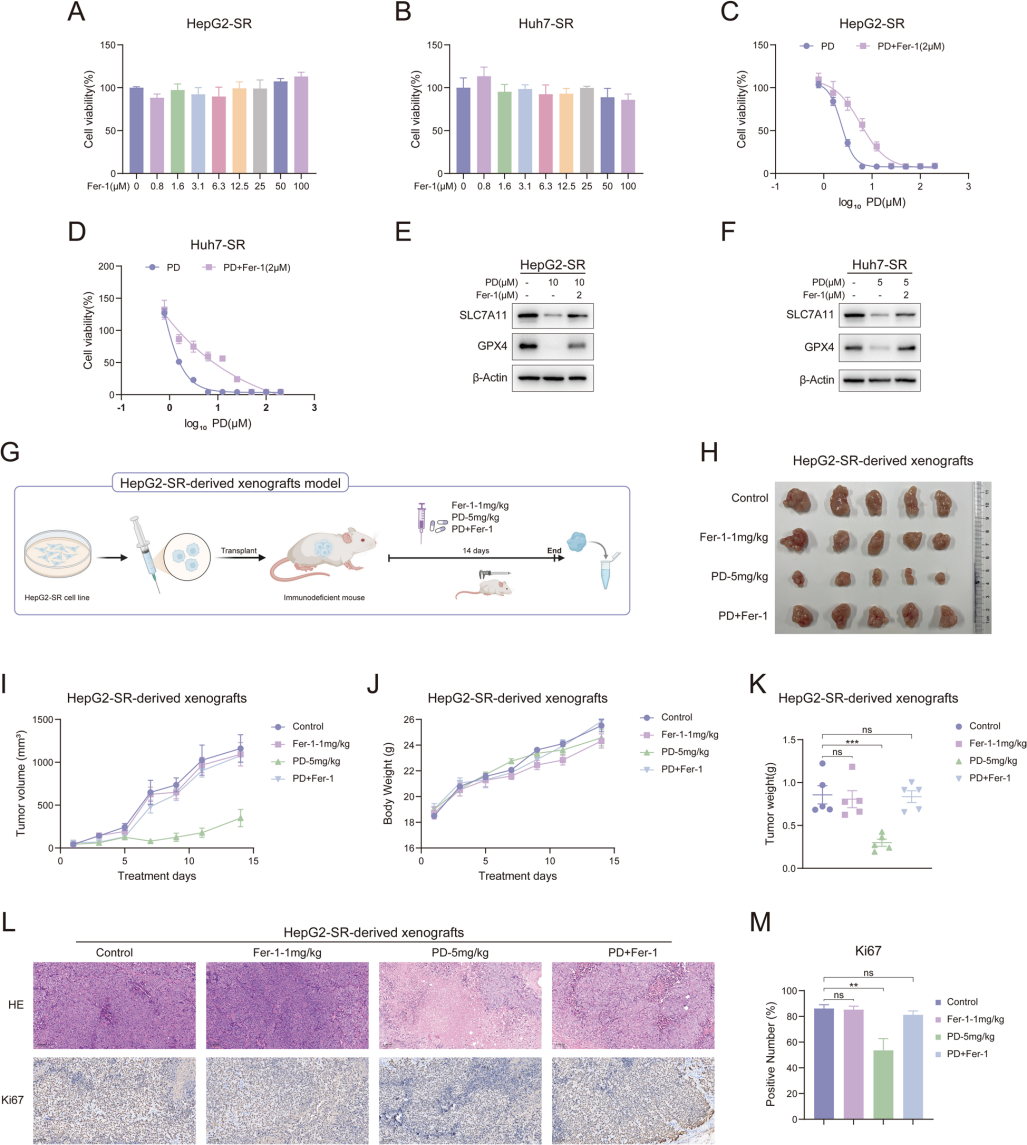
PD inhibits liver cancer progression by inducing ferroptosis. **A**, **B** HepG2-SR and Huh7-SR cells were treated with various concentrations of Fer-1 for 48 h, and cell viability was evaluated by MTT assay (*n* = 3). **C**, **D** HepG2-SR and Huh7-SR cells were treated with various concentrations of PD in the presence of Fer-1 (2 μM) for 48 h, and cell viability was evaluated by MTT assay (*n* = 3). **E**, **F** HepG2-SR and Huh7-SR cells were treated with PD in the presence of Fer-1 (2 μM), and the protein levels of GPX4 and SLC7A11 were measured by western blotting. **G** Schematic diagram of the HepG2-SR-derived xenografts model. **G**-**M** Nude mice bearing HepG2-SR-derived xenografts were intraperitoneally administered PD (5 mg/kg) and Fer-1 (1 mg/kg) every day, *n* = 5. **H** representative images of the tumors, **I**) tumor growth curves, **J** body weight, **K** tumor weight, **L** representative images of HE (Scale bar, 100 μm) and Ki67 (Scale bar, 100 μm) staining in tumor tissues, **M**) positive number of Ki67 (*n* = 5) are shown. Data are presented as mean ± SEM. The p-values were calculated by one-way ANOVAs. *P < 0.05, **P < 0.01, and ***P < 0.001, and ns, not significant

A further validation of PD's induction of ferroptosis was conducted using Ferrostatin-1 (Fer-1), which is an effective and selective inhibitor of ferroptosis [44, 45]. The effect of Fer-1 on the cell viability of HepG2-SR and Huh7-SR cells was first examined, and the results showed that Fer-1 had no impact on cell proliferation (Fig. 7A and B). Based on this, we examined the effects of PD on cells and ferroptosis in the presence of 2 μM Fer-1. As shown in Fig. 7C and D, the inhibitory effect of PD on cell proliferation was diminished in the presence of Fer-1. Similarly, in the presence of Fer-1, the expression levels of GPX4 and SLC7A11 were significantly elevated compared to the group treated with PD alone (Fig. 7E and 7). Furthermore, we conducted in vivo experiments combining PD with Fer-1 to investigate whether PD inhibits tumor progression through ferroptosis. HepG2-SR cells were injected subcutaneously into nude mice to establish a xenograft tumor model (Fig. 7G). The mice were divided into four treatment groups: the control group, Fer-1–1 mg/kg, PD-5 mg/kg, and Fer-1 + PD. After 14 days of treatment, tumor tissues were collected and photographed (Fig. 7H). The tumor volume in the PD-5 mg/kg group was significantly smaller than that in the control group, and the tumor volume in the Fer-1–1 mg/kg group showed no difference from that in the control group (Fig. 7H and I). However, the tumor volumes in the group receiving combined Fer-1 and PD therapy were significantly higher than those in the PD-only group, and showed no difference compared to the control group or the Fer-1-only group (Fig. 7H and I). There were also no significant differences in body weight among the mice in the various groups (Fig. 7J). The results for tumor weight also matched those for tumor volume (Fig. 7K). Hematoxylin and eosin (HE) staining (Fig. 7L upper) and immunohistochemical staining for Ki67 (Fig. 7L lower and M) further confirmed that the ferroptosis inhibitor Fer-1 can limit PD's ability to inhibit tumor growth. Taken together, these results demonstrate that PD induces ferroptosis in sorafenib-resistant liver cancer cells.

### PD increases the concentration of retinoic acid in sorafenib-resistant cells via inhibiting the expression of CYP26A1

**Fig. 8:**
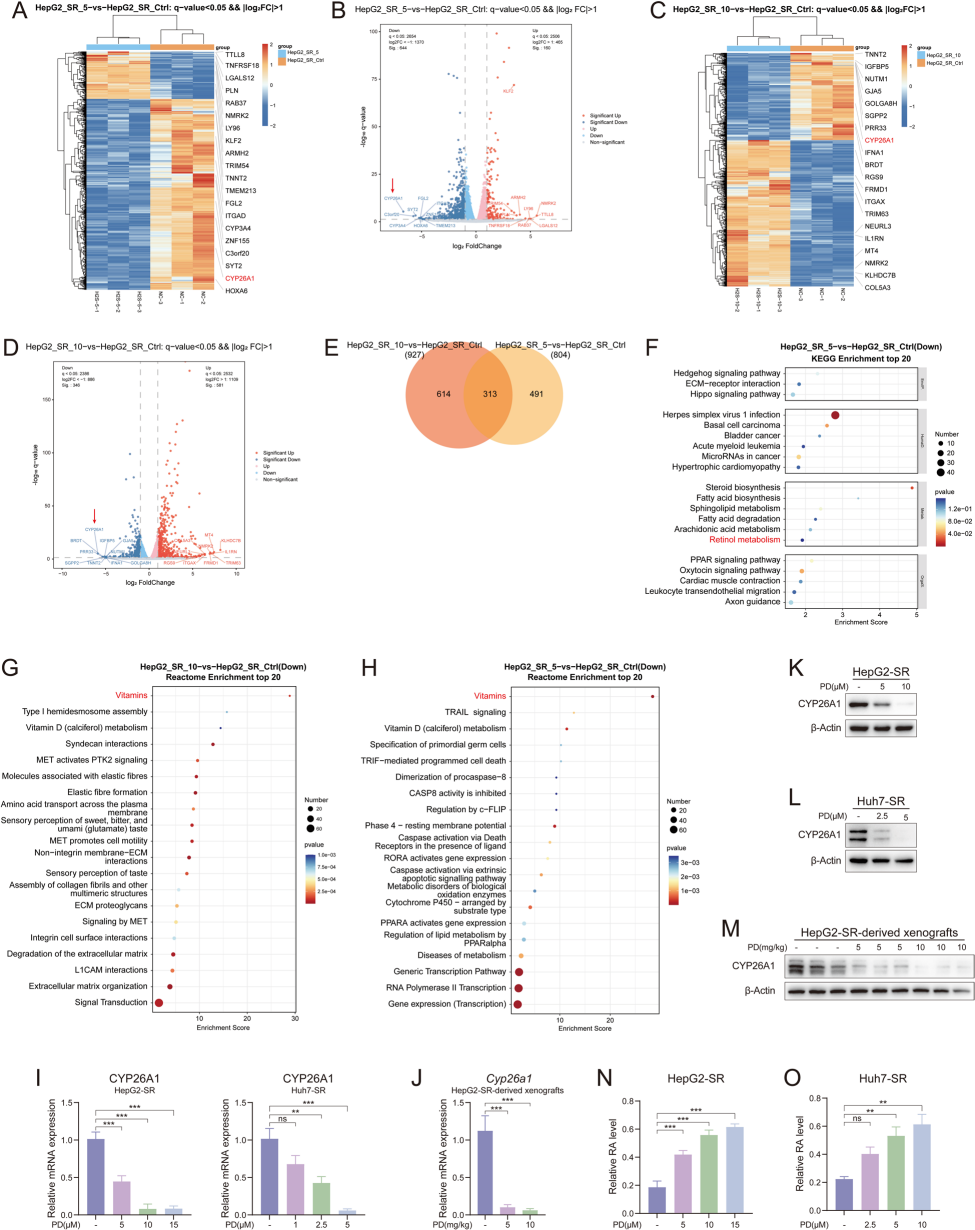
PD increases the concentration of retinoic acid in sorafenib-resistant cells via inhibiting the expression of CYP26A1. **A**-**E** RNA-seq analysis reveals the DEGs in cells from three groups: HepG2-SR-Ctrl, HepG2-SR-5, and HepG2-SR-10. **A** Heatmap representing the significantly regulated genes detected in HepG2-SR-5 compared with HepG2-SR-Ctrl. **B** The volcano plot shows DEGs in HepG2-SR-5 compared with HepG2-SR-Ctrl from the RNA-seq dataset. Red denotes upregulated genes (q < 0.05 and log2 fold change > 1), while blue denotes downregulated genes (q < 0.05 and log2 fold change < −1). The red arrow points to CYP26A1. **C** Heatmap representing the significantly regulated genes detected in HepG2-SR-10 compared with HepG2-SR-Ctrl. **D** The volcano plot shows DEGs in HepG2-SR-10 compared with HepG2-SR-Ctrl from the RNA-seq dataset. The red arrow pointed to CYP26A1. **E** Venn diagram showing the number of significantly different genes in HepG2-SR-5 compared with HepG2-SR-Ctrl and HepG2-SR-10 compared with HepG2-SR-Ctrl. **F** KEGG enrichment analysis of downregulated genes in HepG2-SR-5 compared with HepG2-SR-Ctrl from RNA-seq data. **G** Reactome enrichment analysis of downregulated genes in HepG2-SR-10 compared with HepG2-SR-Ctrl from RNA-seq data. **H** Reactome enrichment analysis of downregulated genes in HepG2-SR-10 compared with HepG2-SR-Ctrl from RNA-seq data. **I** mRNA levels of CYP26A1 in HepG2-SR and Huh7-SR cells. **J** mRNA levels of Cyp26a1 in HepG2-SR-derived xenograft tissues (*n* = 6). **K**-**M** The protein levels of CYP26A1 in K) HepG2-SR, **L** Huh7-SR cells, and **M**) HepG2-SR-derived xenograft tissues were measured by western blotting. **N**, **O** The retinoic acid (RA) levels in **N**) HepG2-SR and **O**) Huh7-SR cells were measured. Data are presented as mean ± SEM. The *p*-values were calculated by one-way ANOVAs. *P < 0.05, **P < 0.01, and ***P < 0.001, and ns, not significant

To uncover the mechanism by which PD induces ferroptosis in sorafenib-resistant cells, we further analyzed the RNA sequencing dataset. After 48 h of PD (5 μM) treatment, 644 genes were downregulated, and 160 genes were upregulated in HepG2-SR cells compared to the control group (Fig. 8A and B). In addition, after treatment with PD (10 μM), 346 genes were downregulated, and 581 genes were upregulated in HepG2-SR cells (Fig. 8C and D). After integrating the differentially expressed genes (DEGs), we performed a cross-analysis of the overlapping genes and found that 313 genes showed expression differences in both the HepG2-SR-5 vs HepG2-SR-ctrl and HepG2-SR-10 vs HepG2-SR-ctrl groups (Fig. 8E). Interestingly, among the 313 differentially expressed genes, CYP26A1 was downregulated in both the HepG2-SR-5 vs HepG2-SR-ctrl and HepG2-SR-10 vs HepG2-SR-ctrl groups (Fig. 8A-D). As a member of the cytochrome P450 family, CYP26A1 maintains dynamic intracellular RA homeostasis by degrading retinoic acid, the active metabolite of vitamin A, through hydroxylation [46]. Consistent with this, KEGG enrichment analysis showed downregulation of the retinol metabolic pathway in HepG2-SR after PD treatment (Fig. 8F). According to Reactome enrichment analysis, Vitamin-related pathways were significantly down-regulated in both 5 μM and 10 μM groups (Fig. 8G and H). RT-qPCR and western blot experiments further confirmed that PD was able to reduce the expression of CYP26A1 in HepG2-SR and Huh7-SR cells (Fig. 8I-L). The same results were also observed in the xenograft tumor model of HepG2-SR (Fig. 8J and M). Following this, we continued to examine whether the content of intracellular retinoic acid changed after the reduction of CYP26A1 expression. As expected, the content of retinoic acid in sorafenib-resistant cells was increased significantly after PD treatment (Fig. 8N and O). These results suggest that PD inhibits the degradation of retinoic acid by decreasing the expression of CYP26A1 and thereby inhibits retinoic acid degradation, leading to retinoic acid accumulation in sorafenib-resistant cells.

### Inhibition of FGFR4 induces ferroptosis and downregulates CYP26A1

**Fig. 9:**
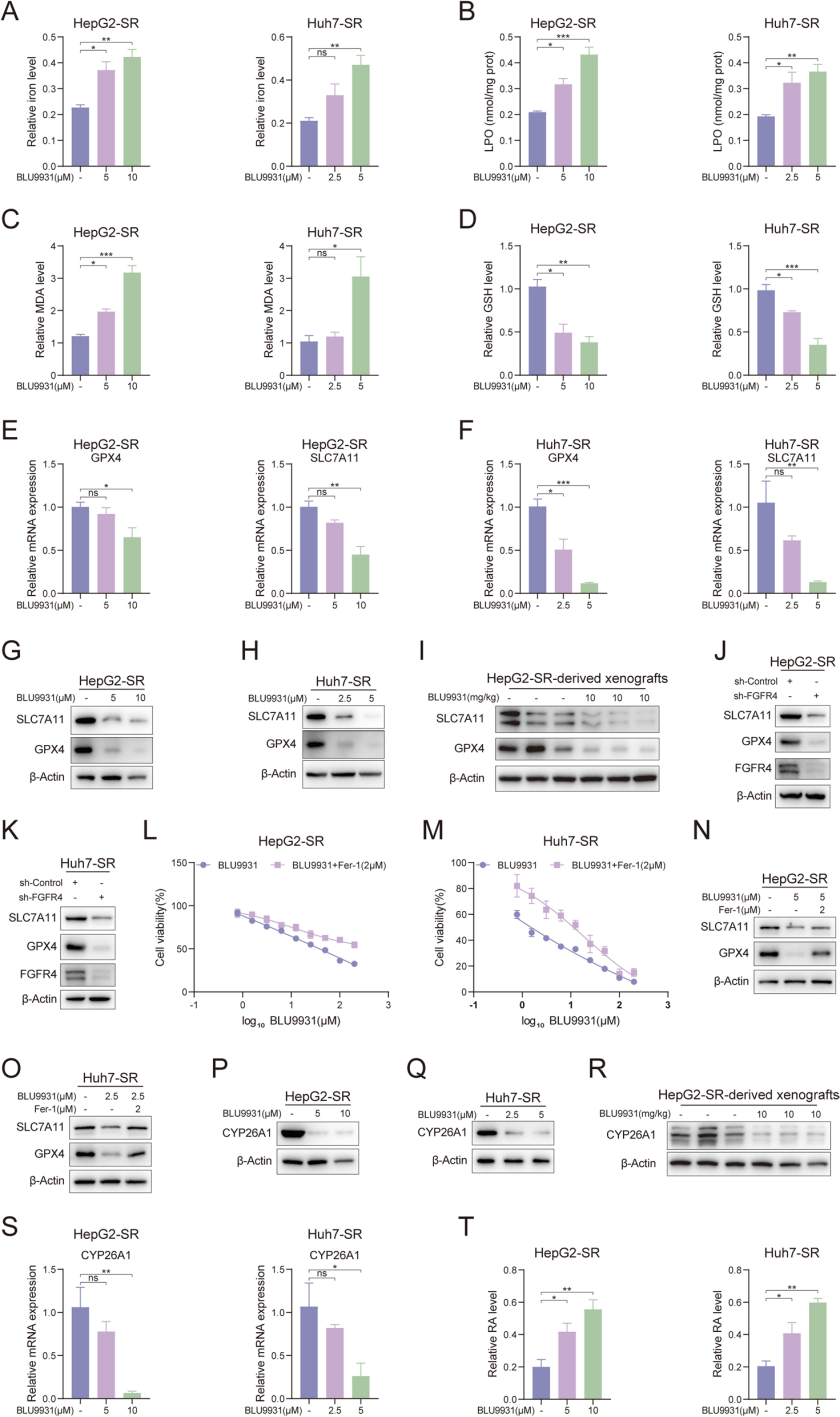
Inhibition of FGFR4 induces ferroptosis and downregulates CYP26A1. **A**-**H** HepG2-SR and Huh7-SR cells were treated with BLU9931 (2.5, 5, and 10 μM) for 48 h. **A** iron level, **B** LPO level, **C** MDA level, **D** GSH, and **E**, **F**) relative mRNA levels of GPX4 and SLC7A11 were measured. The protein levels of GPX4 and SLC7A11 in **G**) HepG2-SR and **H**) Huh7-SR cells were measured by western blotting. **I** The protein levels of GPX4 and SLC7A11 in HepG2-SR-derived xenograft tissues were measured. mRNA levels of Gpx2 and Slc7a11 in HepG2-SR-derived xenografts tissues (*n* = 5). **J**, **K** The protein levels of GPX4 and SLC7A11 were examined in FGFR4-knockdown **J** HepG2-SR and **K**) Huh7-SR cells by western blotting. **L**, **M** HepG2-SR and Huh7-SR cells were treated with various concentrations of BLU9931 in the presence of Fer-1 (2 μM) for 48 h, and cell viability was evaluated by MTT assay (*n* = 3). **N**, **O** HepG2-SR and Huh7-SR cells were treated with BLU9931 in the presence of Fer-1 (2 μM), and the protein levels of GPX4 and SLC7A11 were measured by western blotting. **P**, **Q**, and **S** HepG2-SR and Huh7-SR cells were treated with BLU9931 (2.5, 5, and 10 μM) for 48 h. **S**) Relative mRNA levels of CYP26A1 were measured. The protein levels of CYP26A1 in **P**) HepG2-SR and **Q**) Huh7-SR cells were measured. **R**) The protein expression of CYP26A1 in tumor tissues was shown. **T** The RA levels in HepG2-SR and Huh7-SR cells were measured after treatment with BLU9931. Data are presented as mean ± SEM. The *p*-values were calculated by one-way ANOVAs. *P < 0.05, **P < 0.01, and ***P < 0.001, and ns, not significant

There is no information available on how FGFR4 affects the survival of sorafenib-resistant liver cancer cell lines. Previous experiments have confirmed that FGFR4 is a target of PD. PD increased the content of RA by suppressing the expression of CYP26A1. It is also unknown whether altering FGFR4 activity affects CYP26A1 in sorafenib-resistant liver cancer cell lines. Therefore, we aim to explore whether inhibition of FGFR4 can induce ferroptosis in sorafenib-resistant cells and increase intracellular retinoic acid levels by inhibiting CYP26A1. BLU9931 was used to pharmacologically inhibit FGFR4 in resistant cells. Initially, ferroptosis-related markers Fe^2+^, LPO, MDA, and GSH were detected. As shown in Fig. 9A and B, in HepG2-SR and Huh7-SR cells, BLU9931 upregulated intracellular Fe^2+^ content and increased the levels of LPO. MDA was upregulated in a dose-dependent manner, while GSH was downregulated (Fig. 9C and D). After treatment with BLU9931, the mRNA levels of GPX4 and SLC7A11 were suppressed in HepG2-SR and Huh7-SR cells (Fig. 9E and F). Concurrently, the protein expression levels of GPX4 and SLC7A11 were also suppressed in drug-resistant cell lines and xenograft tumors derived from HepG2-SR cells (Fig. 9G-I). Furthermore, following FGFR4 knockdown, the expression levels of GPX4 and SLC7A11 were also significantly reduced (Fig. 9J and K). Similarly to PD, the ability of BLU9931 to inhibit cell proliferation was reduced in the presence of Fer-1 (Fig. 9L and M). Concurrently, the suppression of GPX4 and SLC7A11 expression by BLU9931 was diminished in the presence of Fer-1 (Fig. 9N and O). These findings indicate that inhibition of FGFR4 can trigger the occurrence of ferroptosis. Next, we measured the expression level of CYP26A1 and the intracellular retinoic acid content. In sorafenib-resistant cell lines, BLU9931 suppressed both mRNA and protein expression of CYP26A1 (Fig. 9P, Q, and S). CYP26A1 was also inhibited in HepG2-SR xenograft tumors (Fig. 9R). As expected, intracellular retinoic acid levels increased when CYP26A1 was inhibited (Fig. 9T). In summary, inhibition of FGFR4 produced similar effects to those observed with PD treatment. This suggests that inhibition of FGFR4 can induce ferroptosis in sorafenib-resistant cells and increase retinoic acid levels by inhibiting CYP26A1.

### Retinoic acid drives ferroptosis in sorafenib-resistant liver cancer cells

**Fig. 10:**
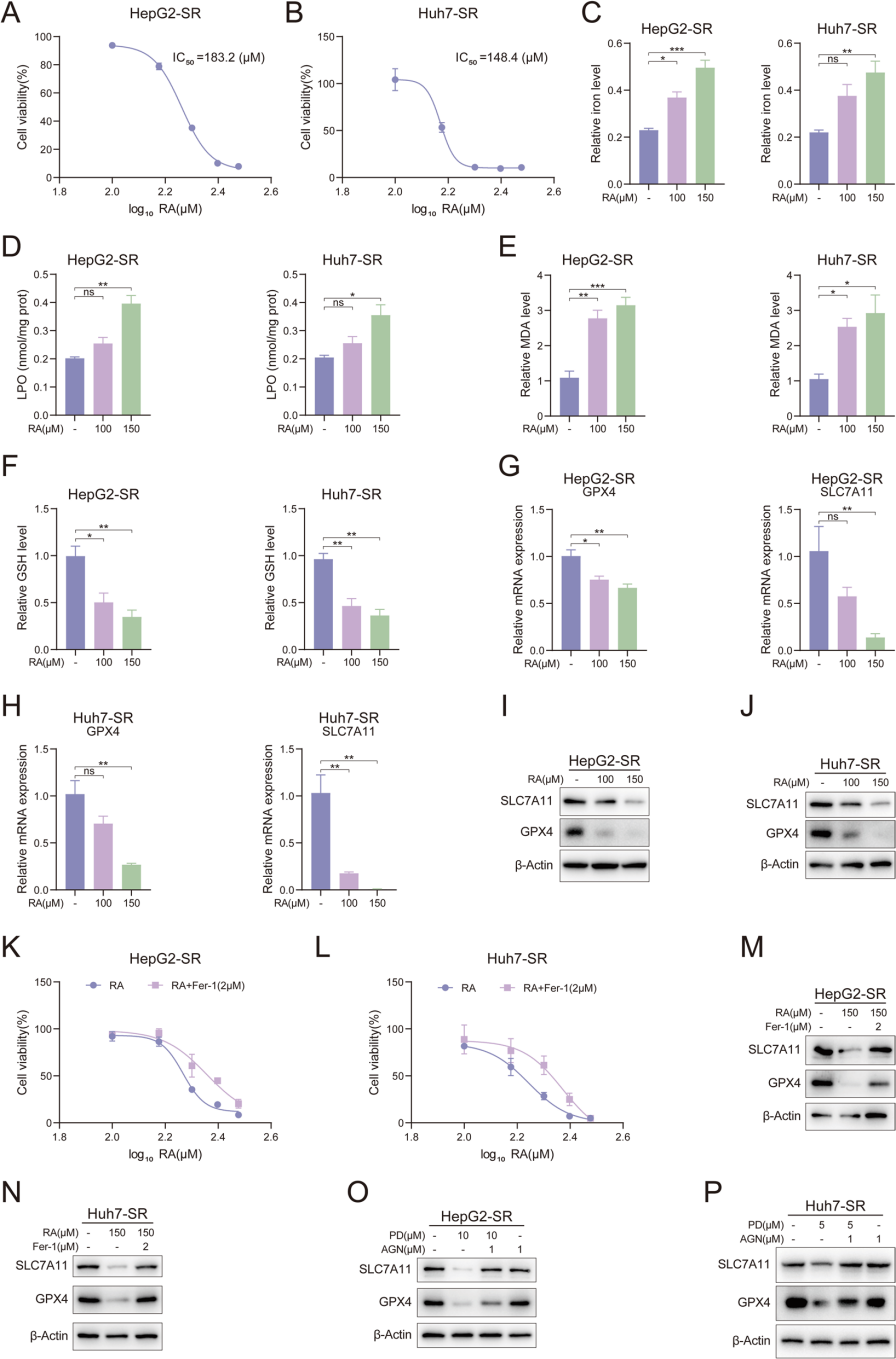
Retinoic acid drives ferroptosis in sorafenib-resistant liver cancer cells. **A**, **B** The viabilities of **A**) HepG2-SR and **B**) Huh7-SR cells were examined after treatment with RA (100 and 150 μM) by MTT assay (*n* = 3). **C**-**J** HepG2-SR and Huh7-SR cells were treated with RA (100 and 150 μM) for 48 h. **C** iron level, **D** LPO level, **E** MDA level, **F** GSH, and **G**, **H**) relative mRNA levels of GPX4 and SLC7A11 were measured. The protein levels of GPX4 and SLC7A11 in I) HepG2-SR and **J**) Huh7-SR cells were measured by western blotting. **K**, **L** HepG2-SR and Huh7-SR cells were treated with various concentrations of RA in the presence of Fer-1 (2 μM) for 48 h, and cell viability was evaluated by MTT assay (*n* = 3). **M**, **N** HepG2-SR and Huh7-SR cells were treated with RA in the presence of Fer-1 (2 μM), and the protein levels of GPX4 and SLC7A11 were measured by western blotting. **O**, **P** HepG2-SR and Huh7-SR cells were treated with PD in the presence of AGN (1 μM), and the protein levels of GPX4 and SLC7A11 were measured by western blotting. Data are presented as mean ± SEM. The p-values were calculated by one-way ANOVAs. *P < 0.05, **P < 0.01, and ***P < 0.001, and ns, not significant

PD induces ferroptosis in sorafenib-resistant liver cancer cells, and inhibition of FGFR4 has the same effect. Both the treatment with PD and the inhibition of FGFR4 suppress CYP26A1 and increase the content of retinoic acid. Therefore, we hypothesize that retinoic acid may be associated with ferroptosis in sorafenib-resistant cells. To confirm this hypothesis, we first investigated the effect of retinoic acid on the proliferation of HepG2-SR and Huh7-SR cells using the MTT assay. As shown in Fig. 10A and B, retinoic acid inhibits the growth of these two cell lines in a dose-dependent manner. The IC_50_ values for retinoic acid in HepG2-SR and Huh7-SR cells, respectively, were 183.2 μM and 148.4 μM. Following exposure to retinoic acid, there was a significant increase in Fe^2+^ concentration in the cells (Fig. 10C). Concurrently, levels of lipid peroxidation markers such as LPO and MDA were also significantly elevated (Fig. 10D and E). Additionally, GSH levels were significantly downregulated by retinoic acid (Fig. 10F). Next, we examined changes in the ferroptosis marker genes GPX4 and SLC7A11. Consistent with expectations, the levels of GPX4 and SLC7A11 in HepG2-SR and Huh7-SR cells were significantly downregulated after retinoic acid treatment (Fig. 10G and H). The results of Western blot experiments also showed that the expression levels of GPX4 and SLC7A11 were significantly reduced (Fig. 10I and J). Similarly, in the presence of Fer-1, the ability of RA to inhibit the proliferation of HepG2-SR and Huh7-SR cells, as well as the extent of reduction in the expression levels of GPX4 and SLC7A11, was significantly diminished (Fig. 10K-N). The previous experimental results demonstrated that PD can increase RA levels and induce ferroptosis, and that RA itself can also induce ferroptosis. Next, we investigated the necessity of RA in the PD-induced ferroptosis process. The expression levels of GPX4 and SLC7A11 were detected in sorafenib-resistant cells treated with PD in the presence of the RA receptor antagonist AGN 193109 (AGN) [47]. The results are shown in Fig. 10O and P; the expression levels of GPX4 and SLC7A11 were significantly reduced when PD was used alone. However, when PD was combined with AGN, the expression levels of GPX4 and SLC7A11 increased compared to PD alone. This is a result of RA receptor blockade, where accumulated retinoic acid cannot exert its effects and consequently fails to induce ferroptosis. These findings demonstrated that RA induces ferroptosis and serves as an essential mediator for PD to trigger this cell death pathway.

## Discussion

In this study, we demonstrated that PD significantly inhibits liver cancer growth both in vitro and in vivo, either as a monotherapy or in combination with sorafenib. Furthermore, PD effectively overcomes sorafenib resistance in preclinical models. Mechanistically, we used a combination of molecular docking and CETSA to robustly confirm that PD directly targets FGFR4. Crucially, we uncovered a previously unrecognized signaling axis wherein PD inhibits FGFR4, leading to the downregulation of CYP26A1 and an increase in retinoic acid (RA). This process ultimately induces ferroptosis, which we identified as a key driver in overcoming resistance. This FGFR4/CYP26A1/RA-ferroptosis cascade represents a novel and treatable target mechanism underlying sorafenib resistance in liver cancer.

Marine-derived compounds have demonstrated significant advantages in pharmaceutical research, with their novel structures and unique biological activities providing important resources for innovative drug development [48]. Metabolic products from marine organisms (such as fungi, actinomycetes, and sponges) possess chemical structures rarely found in terrestrial organisms [49, 50]. The marine natural product marinopyrrole A exhibits antibacterial and antiviral activities [49]. Marine sesquiterpenoid compounds regulate microglial polarization by inhibiting the NF-κB pathway, thereby reducing neuroinflammatory factors (IL-6, TNF-α), offering a new strategy for the development of Alzheimer's disease therapeutics [48]. The Psammaplysene family comprises marine alkaloids with unique structures, primarily isolated from sponges of the genus Psammaplysilla. These sponges are known for producing a variety of tyrosine-derived metabolites that display diverse biological activities [19]. For example, Purpuramine I possesses antibacterial properties [51]. Our research on the biological activities of Psammaplysene D provides a foundation for the development of marine-derived drugs.

Our data clearly demonstrate that PD exhibits potent antitumor activity in multiple liver cancer cell lines that have not developed resistance, and significantly inhibits tumor growth in various in vivo models, particularly when used in synergy with sorafenib to enhance efficacy. This suggests its potential to enhance the efficacy of current first-line treatments. Notably, PD has also demonstrated potent antitumor activity in sorafenib-resistant cell lines and xenograft models. Previous studies have primarily focused on combining the drug with sorafenib to enhance the sensitivity of liver cancer cells to sorafenib, such as doxorubicin [52], 5-fluorouracil [53], and everolimus [54]. However, our study demonstrates that PD not only exhibits synergistic effects when used in combination with sorafenib but also has an independent ability to inhibit the growth of resistant cells. This may reduce the complex side effects associated with using multiple drugs in clinical settings [54, 55]. Over 50% of liver cancer patients develop sorafenib resistance within six months [7, 30], leading to rapid disease progression. The ability of PD to overcome resistance addresses this urgent need.

The direct binding of PD to FGFR4 has been rigorously validated through computational simulations and experimental methods. Molecular docking analysis indicates that PD stably binds to the ATP-binding pocket of the FGFR4 kinase domain [16, 41], with a calculated E-score of −8.47929573, and forms key hydrogen bonds with residues Cys552 [41, 56], Gly556, and Asp630. This binding interaction was functionally validated using a cell thermal stability assay (CETSA), which showed that the thermal stability of FGFR4 in HepG2-SR cells was significantly higher after PD treatment compared to the control group, indicating that PD directly stabilizes the target. At the same time, we also verified that PD binds to FGFR1, FGFR2, and FGFR3 with weaker affinity than to FGFR4. The high consistency between docking predictions and CETSA validation results provides strong evidence that FGFR4 is the target of PD. Furthermore, in both in vitro and in vivo models, the effects of PD were offset when FGFR4 was knocked down or pharmacologically inhibited. This demonstrates that PD exerts its effects through FGFR4, further supporting the conclusion that FGFR4 is the target of PD. BLU9931 is an irreversible covalent inhibitor of FGFR4. As a result of its irreversible binding, it affects the dissociation balance, leading to some side effects such as elevated liver enzymes or gastrointestinal toxicity [57–60]. PD binds to FGFR4 primarily through hydrogen bonds, with a good dissociation constant, which may reduce side effects. Accumulating evidence underscores the multifaceted roles of FGFR4 signaling across various cancers. In hepatocellular and intrahepatic cholangiocarcinoma, FGFR4 promotes tumor progression and therapy resistance through mechanisms including YAP pathway activation and remodeling of the tumor immune microenvironment [18, 61, 62]. Separately, in colon cancer, it facilitates the activation of cancer-associated fibroblasts via the CXCL10-CXCR3 axis [63, 64]. The silencing of FGFR4 potently induces apoptosis even in 5-FU- and oxaliplatin-resistant colorectal cancer models [63, 64]. These findings firmly establish FGFR4 as a pivotal survival factor across cancer types, highlighting its broader implications in mediating therapeutic resistance. Therefore, PD, a marine-derived compound, can serve as a lead compound for the development of new FGFR4 inhibitors.

Ferroptosis, as a form of cell death, plays an important role in the treatment of various diseases [65–68], particularly in tumors [69–71]. During ferroptosis, the abnormal accumulation of ferrous ions (Fe^2^⁺) first triggers a surge of reactive oxygen species (ROS), which then attack polyunsaturated fatty acids (PUFAs) in the cell membrane, leading to a chain reaction of lipid peroxidation (LPO) and the generation of the toxic product malondialdehyde (MDA) [72]. Concurrently, the exhaustion of glutathione (GSH), a key antioxidant, weakens the cell's reductive capacity, leading to the inactivation of glutathione peroxidase 4 (GPX4)-the core enzyme responsible for clearing lipid peroxidation [42]. The dysfunction of GPX4 further accelerates the uncontrolled spread of LPO [73]. The primary cause of this vicious cycle is directly related to the functional inhibition of the cystine/glutamate reverse transporter SLC7A11: when SLC7A11 is downregulated, cellular uptake of cystine is obstructed, preventing the synthesis of sufficient GSH, ultimately leading to the complete collapse of the antioxidant system [72]. Based on RNA-seq results, we found that inhibiting FGFR4 using PD or BLU9931 induces ferroptosis in sorafenib-resistant liver cancer cells, and this conclusion is confirmed by characteristic biochemical changes. Notably, FGFR4 inhibition significantly increased intracellular iron levels, lipid peroxidation (LPO), and malondialdehyde (MDA), while glutathione (GSH) levels decreased by nearly 50% compared to the control group. Meanwhile, the expression of GPX4 and SLC7A11 was downregulated at both the mRNA and protein levels, confirming that the key ferroptosis defense system was inhibited. These data indicate that inhibition of FGFR4 disrupts oxidative-reductive balance by depleting antioxidant reserves (GSH) and inactivating GPX4, thereby allowing lethal lipid peroxidation. Previous reports have shown that lenvatinib can induce ferroptosis by inhibiting FGFR4, leading to the alleviation of liver cancer [74]. Moreover, the imidazole derivative F30, which targets FGFR4, upregulates the expression of heme oxygenase 1 (HMOX1), promoting ferroptosis in liver cancer cells [75]. Zou et al. found that inhibiting FGFR4 can overcome resistance in recalcitrant HER2-positive breast cancer, while also uncovering synergistic effects between anti-FGFR4 and anti-HER2 therapies in breast cancer with intrinsic or acquired resistance [76]. However, there have been few previous reports on the role of FGFR4 in sorafenib-resistant liver cancer. Our study highlights that inhibiting FGFR4 promotes ferroptosis, offering new insights and directions for the treatment of sorafenib-resistant liver cancer.

Retinoic acid is an active derivative of vitamin A, which acts as a ligand to bind with nuclear receptors (RAR/RXR), activating target gene transcription and regulating cell differentiation, proliferation, and apoptosis [77, 78]. CYP26A1 belongs to the cytochrome P450 superfamily (CYP26 family), whose primary function is to catalyze the metabolic inactivation of retinoic acid (RA). CYP26A1 catalyzes hydroxylation reactions (such as 4-hydroxylation or 18-hydroxylation) to convert the active form of all-trans retinoic acid (ATRA) into polar metabolites, thereby reducing its biological activity [79, 80]. This process regulates intracellular RA concentrations through a negative feedback mechanism, preventing cellular toxicity or developmental abnormalities caused by excessive RA accumulation [46, 81]. Reported research indicates that metformin can reduce the development of liver cancer by downregulating CYP26A1 expression [82]. Additionally, retinoic acid metabolism blocking agents targeting CYP26A1 activity have been developed [83]. In the present study, transcriptomic analysis of HepG2-SR cells showed that CYP26A1 was the most significantly downregulated gene (log2FC = −5.9 and −6.7) after treatment with two doses of PD, suggesting that RA levels may increase in resistant cells following PD treatment. As expected, consistent with the inhibition of CYP26A1, RA levels were significantly elevated in drug-resistant cells treated with PD or BLU9931. Importantly, ferroptosis occurred in sorafenib-resistant hepatic carcinoma cells when treated with RA. It was also found that RA is an essential mediator in the PD-induced program of ferroptosis. Previous studies have shown that all-trans retinoic acid can induce high expression of ATG7 and autophagy in liver cancer cells [84]. Sun et al. reported that RA can regulate ferroptosis to inhibit the migratory ability of liver cancer cells [85]. RA can also enhance the sensitivity of liver cancer cells to sorafenib by regulating the PAK1 or AMPK signaling pathways [86, 87]. In this study, we propose for the first time that RA, which increases due to the inhibition of CYP26A1, can trigger ferroptosis in sorafenib-resistant liver cancer cells.

In summary, our data collectively reveal a coherent signaling pathway in sorafenib-resistant liver cancer wherein PD binding to FGFR4 suppresses CYP26A1 transcription, resulting in retinoic acid (RA) accumulation that ultimately triggers ferroptosis. Here, PD exhibits its potent antitumor potential by targeting FGFR4. CYP26A1, as a metabolic gatekeeper protein, prevents RA accumulation, while RA directly executes ferroptosis through iron-dependent lipid peroxidation.

## Conclusions

To our knowledge, we have for the first time revealed that the marine-derived compound Psammaplysene D possesses anti-liver cancer activity, and Psammaplysene D could serve as a lead compound for potential anticancer drug development. This provides new insights and strategies for the development of marine drugs. Furthermore, this is the first demonstration that pharmacological FGFR4 inhibition can directly induce ferroptosis in sorafenib-resistant liver cancer cells. We further elucidated the previously unrecognized role of CYP26A1/RA in ferroptosis regulation. Most importantly, we have defined the FGFR4/CYP26A1/RA-ferroptosis axis as a drug target specific to sorafenib resistance, providing a mechanism-based foundation for overcoming treatment failures in drug-resistant liver cancer.

## Supplementary Information

Supplementary Material 1.

Supplementary Material 2.

## Data Availability

The datasets used and/or analyzed during the current study are available from the corresponding author on reasonable request. The data supporting our conclusion were also obtained from the PDB database (https://www.rcsb.org/).

## Abbreviations

GPX4: Glutathione peroxidase 4
CYP26A1: Cytochrome P450 family 26 subfamily a member 1
FGFR4: Fibroblast growth factor receptor 4
SLC7A11: Solute carrier family 7a member 11
LPO: Lipid peroxidation
MDA: Malondialdehyde
GSH: Glutathione
RA: Retinoic acid
CETSA: Cellular thermal shift assay
HE: Hematoxylin–eosin
MTT: Thiazolyl blue tetrazolium bromide
