# Supplementary material for: Psammaplysene D overcomes sorafenib resistance in liver cancer by targeting FGFR4/CYP26A1-retinoic acid axis to drive ferroptosis
Source: J Exp Clin Cancer Res. 2026 Jan 6;45:44. doi: 10.1186/s13046-025-03622-1 (PMC12888168; doi:10.1186/s13046-025-03622-1)
Supplement: Supplementary file 2 — Supplementary Material 2. [file 13046_2025_3622_MOESM2_ESM.docx]

Supplementary Table 1 The sequences of siRNA

| siRNA | Sequence (5’-3’) |
| --- | --- |
| FGFR4-1 | GGCUGAAGCACAUCGUCAUTT |
| FGFR4-2 | CCAGGUAUACGGACAUCAUTT |
| NC | UUCUCCGAACGUGUCACGUTT |

Supplementary Table 2 The sequences of shRNA

| shRNA | Sequence (5’-3’) |
| --- | --- |
| pGPU6/GFP/Neo | TTGTTTGACCGGGTGTACACA |

Supplementary Table 3 The information of plasmid

| name | plasmid vector |
| --- | --- |
| OE-FGFR4 | PLVX-puro |

Supplementary Table 4 The sequences of primer

| Gene | Forward sequence (5’-3’) | Reverse sequence (5’-3’) |
| --- | --- | --- |
| β-Actin | GATGAGATTGGCATGGCTTT | GTCACCTTCACCGTTCCAGT |
| GPX4 | ATCGACGGGCACATGGTTAA | AACCACACTCAGCGTATCGG |
| SLC7A11 | TCTCCAAAGGAGGTTACCTGC | AGACTCCCCTCAGTAAAGTGAC |
| CYP26A1 | CTCTTCCTGGCTGCGATCA | TACCATCTGCAAGGTTTCCCC |


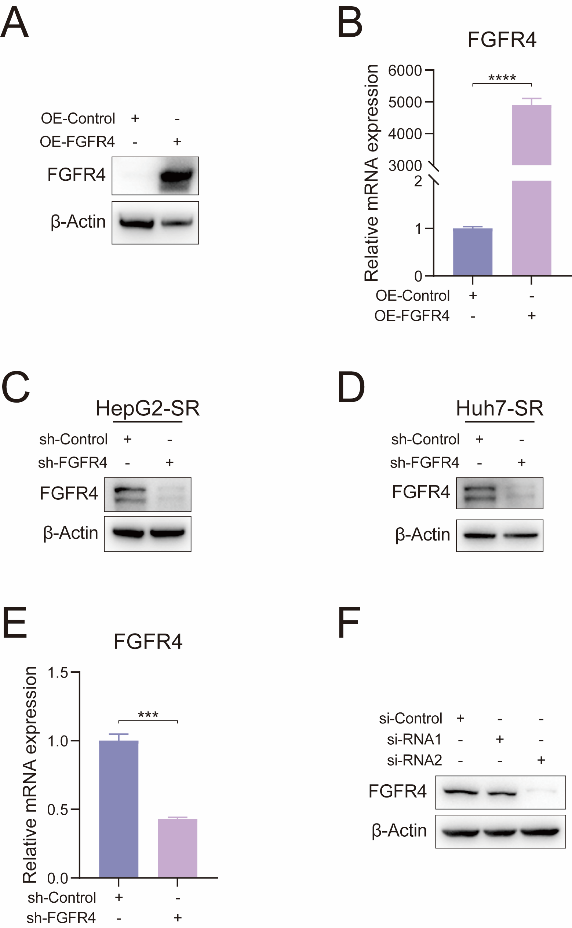


Supplementary Figure 1. A, B) FGFR4 was overexpressed in HepG2-SR cells, and both the A) protein and B) mRNA levels of FGFR4 were detected. C-E) FGFR4 was knocked down using shRNA in HepG2-SR and Huh7-SR cells. C, D) FGFR4 protein levels were detected in both cell lines. E) mRNA levels were detected in HepG2-SR cells. F) FGFR4 protein levels were detected in HepG2-SR cells following siRNA-mediated knockdown of FGFR4.


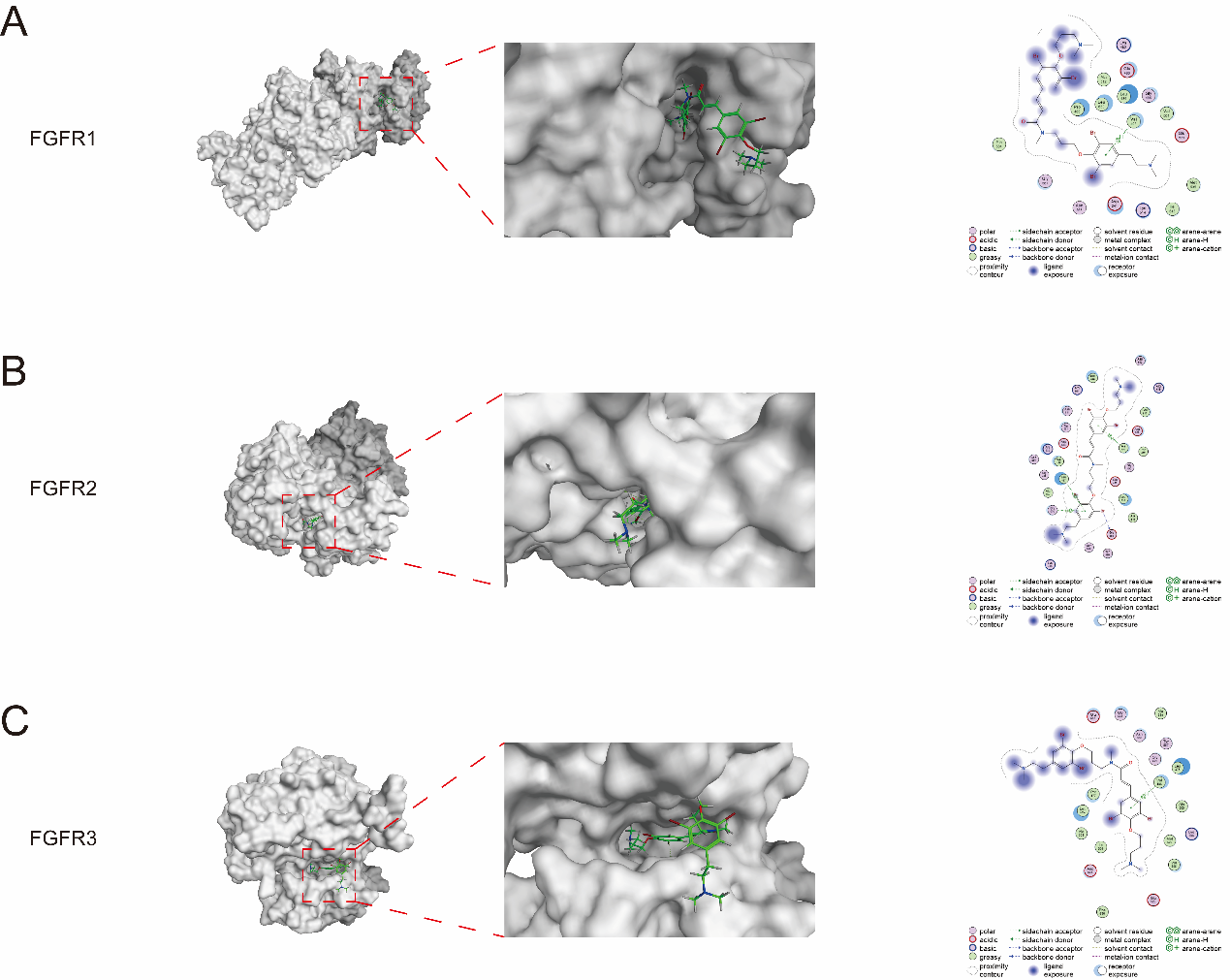


Supplementary Figure 2. A) Molecular docking was performed to predict the binding region between PD and FGFR1, A left) the docking configuration, and A right) interaction forces are displayed. B) Molecular docking was performed to predict the binding region between PD and FGFR2, B left) the docking configuration, and B right) interaction forces are displayed. C) Molecular docking was performed to predict the binding region between PD and FGFR3, C left) the docking configuration, and C right) interaction forces are displayed.
