# Supplementary material for: Psammaplysene D overcomes sorafenib resistance in liver cancer by targeting FGFR4/CYP26A1-retinoic acid axis to drive ferroptosis
Source: J Exp Clin Cancer Res. 2026 Jan 6;45:44. doi: 10.1186/s13046-025-03622-1 (PMC12888168; doi:10.1186/s13046-025-03622-1)
Supplement: Supplementary file 1 — Supplementary Material 1. [file 13046_2025_3622_MOESM1_ESM.docx]

Original western blots

Fig. 4K

p-FGFR4

170KDa

70KDa

FGFR4

170KDa

70KDa

β-Actin

55KDa

40KDa

Fig. 4L

p-FGFR4

70KDa

170KDa

FGFR4

170KDa

70KDa

β-Actin

55KDa

40KDa

Fig. 4M

FGFR4

170KDa

70KDa

β-Actin

55KDa

40KDa

Fig. 4P

FGFR1

170KDa

70KDa

β-Actin

40KDa

55KDa

Fig. 4Q

FGFR2

170KDa

70KDa

β-Actin

40KDa

55KDa

Fig. 4R

FGFR3

70KDa

170KDa

β-Actin

55KDa

40KDa

Fig. 6L

SLC7A11

55KDa

40KDa

GPX4

15KDa

β-Actin

40KDa

Fig. 6M

SLC7A11

55KDa

40KDa

GPX4

15KDa

β-Actin

40KDa

Fig. 6N

SLC7A11

70KDa

55KDa

40KDa

GPX4

15KDa

25KDa

β-Actin

35KDa

40KDa

Fig. 7E

SLC7A11

70KDa

55KDa

40KDa

GPX4

35KDa

25KDa

15KDa

β-Actin

55KDa

40KDa

35KDa

Fig. 7F

SLC7A11

55KDa

40KDa

70KDa

GPX4

35KDa

25KDa

15KDa

β-Actin

55KDa

40KDa

35KDa

Fig. 8K

CYP26A1

70KDa

55KDa

β-Actin

40KDa

35KDa

Fig. 8L

CYP26A1

70KDa

55KDa

40KDa

β-Actin

35KDa

40KDa

Fig. 8M

CYP26A1

70KDa

55KDa

β-Actin

35KDa

40KDa

Fig. 9G

SLC7A11

55KDa

GPX4

15KDa

β-Actin

35KDa

40KDa

Fig. 9H

SLC7A11

55KDa

GPX4

25KDa

15KDa

β-Actin

35KDa

40KDa

Fig. 9I

SLC7A11

40KDa

55KDa

70KDa

GPX4

15KDa

25KDa

β-Actin

35KDa

40KDa

Fig. 9J

SLC7A11

55KDa

GPX4

15KDa

25KDa

FGFR4

130KDa

70KDa

β-Actin

40KDa

35KDa

Fig. 9J

SLC7A11

55KDa

GPX4

25KDa

15KDa

FGFR4

130KDa

70KDa

β-Actin

40KDa

35KDa

Fig. 9N

SLC7A11

40KDa

70KDa

55KDa

GPX4

25KDa

15KDa

β-Actin

55KDa

35KDa

40KDa

Fig. 9O

SLC7A11

55KDa

70KDa

GPX4

15KDa

25KDa

β-Actin

55KDa

40KDa

Fig. 9P

CYP26A1

55KDa

β-Actin

35KDa

40KDa

Fig. 9Q

CYP26A1

70KDa

55KDa

β-Actin

40KDa

Fig. 9R

CYP26A1

70KDa

55KDa

β-Actin

40KDa

Fig. 10I

SLC7A11

55KDa

GPX4

15KDa

β-Actin

40KDa

35KDa

Fig. 10J

SLC7A11

55KDa

GPX4

15KDa

β-Actin

35KDa

40KDa

Fig. 10M

SLC7A11

40KDa

55KDa

GPX4

25KDa

15KDa

β-Actin

55KDa

35KDa

40KDa

Fig. 10N

SLC7A11

40KDa

70KDa

55KDa

GPX4

25KDa

15KDa

β-Actin

55KDa

40KDa

35KDa

Fig. 10O

SLC7A11

40KDa

55KDa

GPX4

25KDa

15KDa

β-Actin

55KDa

35KDa

40KDa

Fig. 10P

SLC7A11

70KDa

40KDa

55KDa

25KDa

GPX4

15KDa

β-Actin

70KDa

55KDa

40KDa
